# 2D Conductive MOFs Intercalated in MXene Interlayer for Fast and Trace Detection of Triethylamine at Room Temperature

**DOI:** 10.1002/advs.202500786

**Published:** 2025-05-24

**Authors:** Hao Zhang, Wei Cao, Jingfeng Wang, Lei Guo, Pu‐Hong Wang, Zhi‐Jun Ding, Lingmin Yu

**Affiliations:** ^1^ State Key Laboratory of Chemistry for NBC Hazards Protection BeiJing 102205 China; ^2^ Xi'an Technological University School of Materials and Chemical Engineering Xi'an Shaanxi 710021 China

**Keywords:** Cu‐HHTP, MXene, room temperature sensing, triethylamine

## Abstract

Metal–organic frameworks (MOFs) are newly developed materials for gas sensing applications currently. However, the prolonged response time limits their future applications because of their poor electrical conductivity. In this context, alternating stacked MXene@Cu‐HHTP heterostructures characterized by a sandwich‐type architecture comprised of Cu‐HHTP (copper‐catecholate frameworks), 2D conductive MOFs, and layered MXene achieve high‐performance triethylamine (TEA) sensing. The unique interlayer pore architecture within the MXene@Cu‐HHTP composites facilitates efficient mass transfer of gas molecules while retaining the large surface area and porosity characteristics of the MOFs, leading to rapid TEA response. MXene@Cu‐HHTP composites respond to 50 ppm TEA in only 4 s and low detection limit (1 ppm). Demonstrated higher sensitivity compared to the original Cu‐HHTP sensor (≈21 times at 200 ppm TEA). At room temperature and atmospheric conditions, the value of moisture resistance of MXene@Cu‐HHTP composites can reach 80% through continuous real‐time dynamic testing.

## Graphical abstract

This study presents alternating MXene@Cu‐HHTP (HHTP = 2,3,6,7,10,11‐hexahydroxytriphenylene) heterostructures with a sandwich‐like architecture, combining 2D conductive metal–organic frameworks (2D‐cMOFs) and Ti_3_C_2_T_X_ MXene to enhance triethylamine (TEA) sensing. The composite achieves rapid response (4 s at 50 ppm), ultralow detection limit (1 ppm), 21‐fold sensitivity improvement (200 ppm) over pure Cu‐HHTP, and 80% humidity resistance.

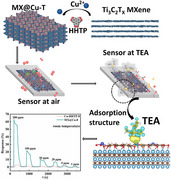

## 1 Introduction

Triethylamine (TEA) is a volatile, flammable, and toxic compound, which can irritate the mucous membranes and central nervous system, leading to significant dysfunction of both the liver and the nervous system.^[^ ^1^, ^2^ ^]^ The National Institute for Occupational Safety and Health (NIOSH) and the American Conference of Government Industrial Health Workers (ACGIH) recommend that workplace triethylamine concentrations should not be higher than 10 and 1 ppm, respectively.^[^ ^3^, ^4^ ^]^ In addition, the food spoilage process releases TEA,^[^ ^5^, ^6^ ^]^ and some research employs the concentration of TEA as a measure of their freshness.^[^ ^7^, ^8^, ^9^, ^10^ ^]^ Thus, the development of reliable and real‐time methodologies for the detection of trace TEA levels has become urgently imperative.

At present, chemical resistance gas sensor is one of the effective methods to detect TEA,^[^ ^11^, ^12^ ^]^ as it is compact, portable, and low cost. The existing TEA gas sensitive detection materials are mainly Metal Oxide Semiconductors (MOSs) such as CuO,^[^ ^13^ ^]^ ZnO,^[^ ^14^ ^]^ In_2_O_3_,^[^ ^15^ ^]^ and SnO_2_,^[^ ^16^ ^]^ which have been systematically investigated and extensively utilized. Although the response value is high, high operating temperature, long response time, poor selectivity, and large environmental impact (e.g., humidity) still need to be overcome to expand their practical application.

In the realm of gas detection, metal–organic frameworks (MOFs) have been regarded as promising candidates lately due to their porous structure, extensive surface area, and tunable architecture. However, their gas‐sensing employment is hampered by the low electrical conductivity and inferior gas‐sensitivity performance, such as poor stability in humidity.^[^ ^17^ ^]^ Very recently, 2D conductive metal–organic frameworks (2D‐cMOFs) with enhanced electron transfer efficiency and inherent porous character for guest molecular interactions have been developed, leading to the elevation of gas sensing performance by introducing large π‐conjugated ligands.^[^ ^18^, ^19^, ^20^ ^]^ Current studies have demonstrated that combining primitive 2D‐cMOFs with conductive materials, including carbon‐based materials and conductive polymers, can effectively improve the electronic conductivity of MOFs, thereby improving performance stability and enhancing selectivity.^[^ ^21^, ^22^ ^]^ Lim et al.^[^ ^23^ ^]^ reported a hybrid structure of Cu_3_HHTP_2_ (HHTP = 2,3,6,7,10,11‐hexahydroxytriphenylene), 2D‐cMOF and laser‐induced graphene (LIG) for high‐performance NO_2_ sensing. LIG@Cu_3_HHTP_2_ is capable of realizing fast response/recovery to NO_2_ (16 s/15 s), and low detection limit (theoretical value: 0.168 ppb) at room temperature. PN heterojunction formed through Cu_3_(HHTP)_2_ material in situ onto SnS_2_ nanolayers have been synthesized by Huang et al,^[^ ^24^ ^]^ thereby promoting disgusting performance NH_3_ sensing with four times higher response and low detection limit (experimental value: 125 ppb; theoretical value: 9.84 ppb). Liu et al.^[^ ^25^ ^]^ applied physiological coagulation to attach ZnO nanoarrays to alginate fibers (AF), and introduced HHTP into ZnO epitaxy to synthesis AF/ZnO/Zn_3_(HHTP)_2_. This sensor has a 0.5 ppm TEA low limit detection at 50℃ with a response/recovery time of ≈56 s/128 s.

MXene, characterized by its exceptional electrical conductivity and rich surface chemistry, presents a promising opportunity for the construction of 2D heterostructures.^[^ ^26^ ^]^ The accessible surface area of MXene is ample to supply active sites for the insertion and extraction of metal ions.^[^ ^27^ ^]^ From this standpoint, designing heterostructures between MOFs with MXene not only mitigates the significant aggregation issues associated with nanosheets but also augments electrical conductivity, thereby facilitating efficient charge transport.^[^ ^28^ ^]^ However, MXene‐based conductive materials have rarely been introduced into 2D‐cMOFs to improve TEA gas sensing performance.

Here, we introduce 2D Ti_3_C_2_T_X_ MXene (T_x_ represents the surface terminal groups like ─F, ─O, or ─OH) sheets as a growth platform for Cu‐HHTP to achieve real‐time monitoring of ppm‐grade TEA. Cu‐HHTP can be self‐assembled on the surface of MXene owing to the abundant nucleation sites such as ─F, ─O, and ─OH groups and form sandwich‐like macroscopic structures. The in‐situ growth of 2D‐cMOFs on MXene substrates effectively amplifies the inherent advantages of the MOFs, while simultaneously addressing their chemical instability and low conductivity. Therefore, the MXene@Cu‐HHTP composite structure exhibits ultra‐short response times (τ_res_ = 4 s, τ_rec_ = 187 s at 50 ppm TEA) and a lower detection limit (1 ppm) with higher sensitivity (≈21 times at 200 ppm TEA) compared to the original Cu‐HHTP sensor at room temperature and atmospheric environmental conditions. Finally, a MXene@Cu‐HHTP electronic TEA gas sensor for food spoilage real‐time detection is successfully constructed.

## 2 Results and Discussion

### 2.1 Structure and Composition Characterization

**Figure 1:**
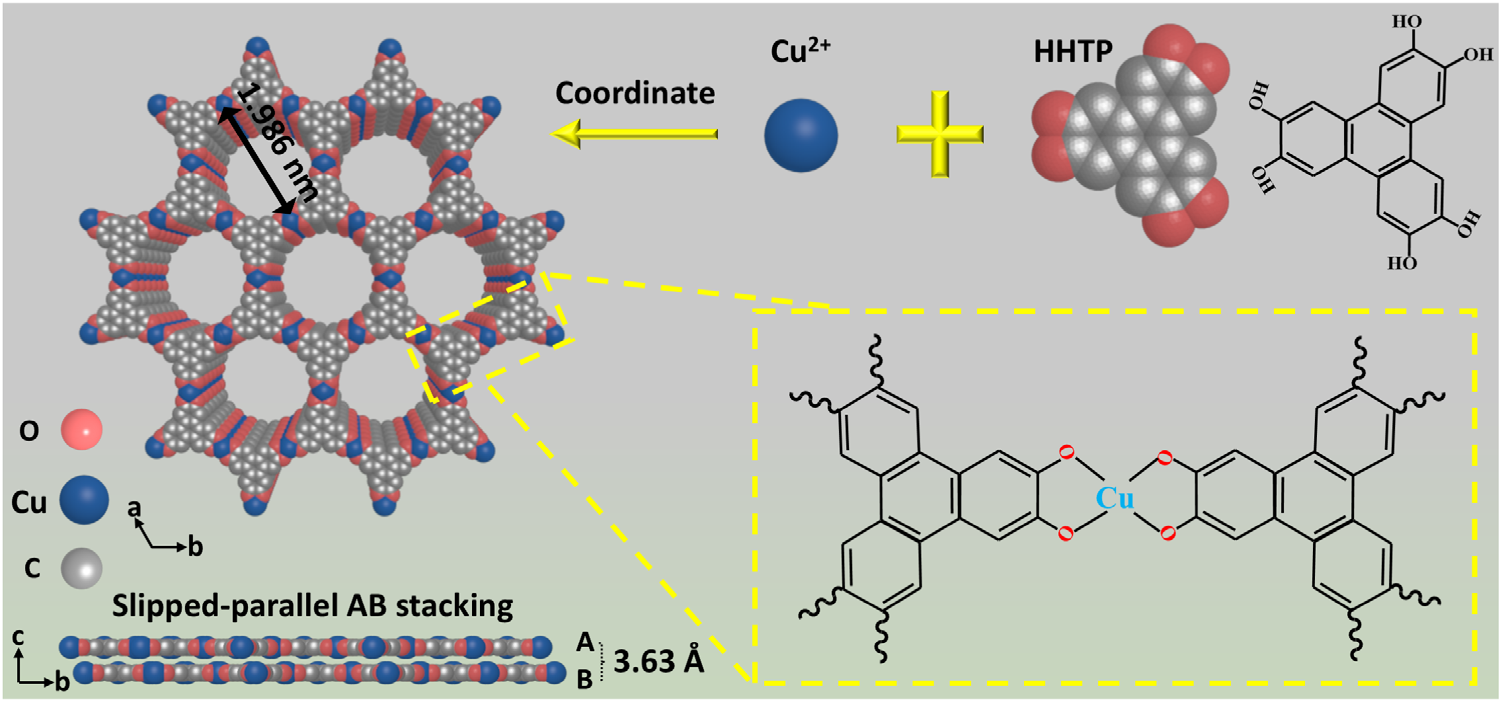
Schematic illustration of synthetic Cu‐HHTP.

**Figure 2:**
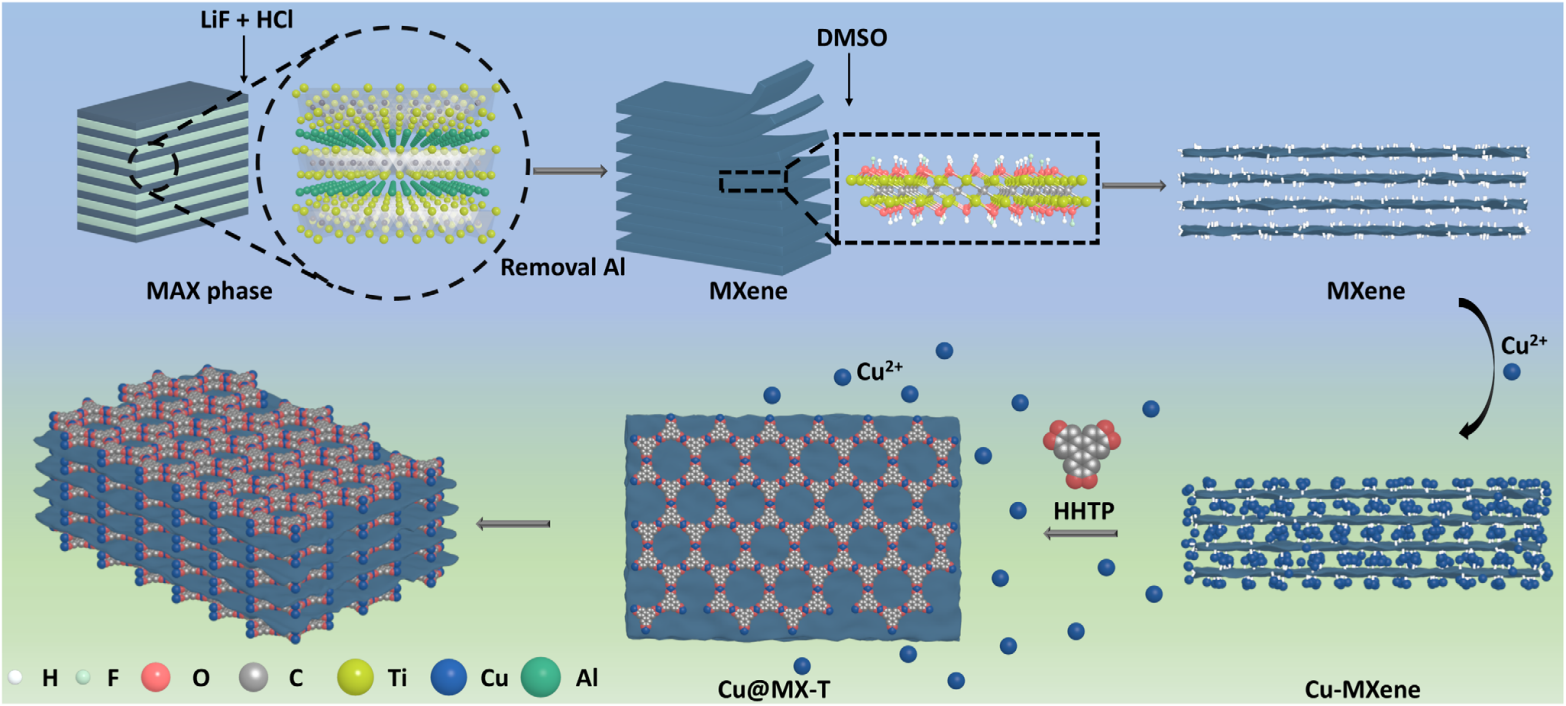
Schematic illustration of synthetic MX@Cu‐T.

**Figure 3:**
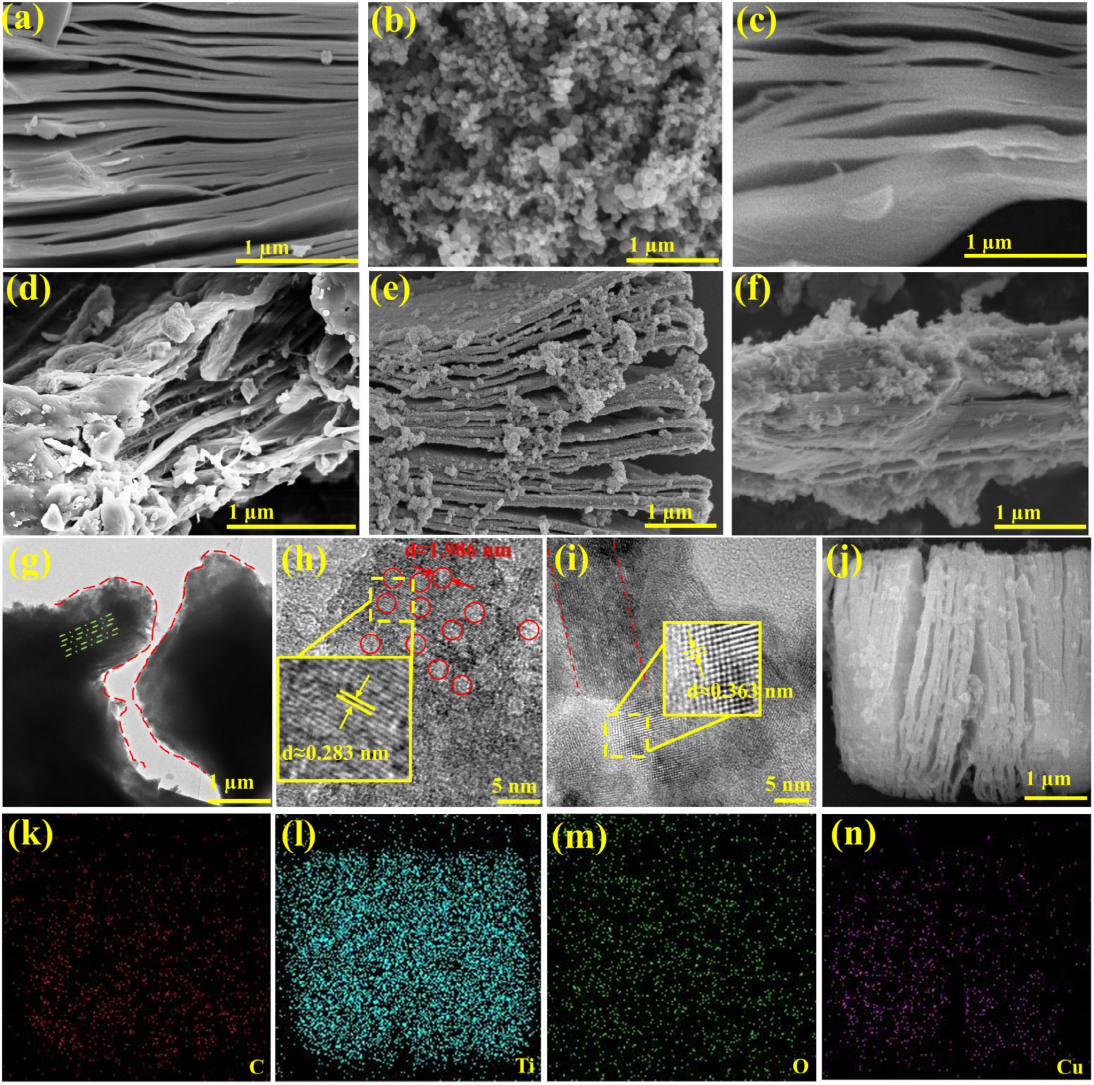
Scanning electron microscopy (SEM) images of a) MXene, b) Cu‐HHTP‐8, c) Cu‐MXene, d) MX@Cu‐2, e) MX@Cu‐8, f) MX@Cu‐16, g) TEM image, h,i) HRTEM image, j) Elemental mapping of k) C, l) Ti, m) O, n) Cu.

The structural unit of the Cu‐HHTP comprises a Cu metal node and the coordinating organic ligand of HHTP, as schematic illustrated in **Figure** **1**. The strong charge separation domain, combined with effective orbital overlap, bestows Cu‐HHTP with remarkable electronic transport properties and facilitates the implementation of gas sensing technologies with reduced power consumption. As depicted in Figure 3b, it can be observed that the amorphous Cu‐HHTP nano‐particles materials bonded with each other have a chaotic and irregular overall structure, relatively poor specific surface area and few active sites. The microstructure of MXene is observed by scanning electron microscopy (SEM). Typical “accordion” multi‐layered structure with clear gaps within the Ti_3_C_2_T_x_ nanosheets can be observed in Figure S1 (Supporting Information), indicating that the Al layer has been etched. As is shown in Figure 3a, layer space of MXene becomes larger via the pretreatment of DMSO solution. Cu^2+^ is introduced into copper acetate solution to bind with rich functional groups (─OH, ─O, ─F, etc.) on the surface of MXene (Figure 3c; Figure S2, Supporting Information), where Cu^2+^ is then coordinated with HHTP ligand to form hexagonal layers on the MXene plane and contribute to an extended 2D structure (**Figure** **2**).

Numerous functional groups are present on the surface of these layers, which play a pivotal role in the effective capture of gas molecules. The introduction of MXene material gives Cu‐HHTP growth space, and the XRD shift to a small angle shows that the more obvious nano amorphous Cu‐HHTP grows between MXene layers (Figure 2a), resulting in a larger MXene layer spacing. Cu‐HHTP also grows around and wraps the MXene lamellar surface, increasing the specific surface area. **Figure** **3**d shows the SEM of MX@Cu‐2, revealing several Cu‐HHTP nanoparticles growing on MXene. With the increase of reaction time, the growth of amorphous materials of Cu‐HHTP nanoparticles attached to the surface of MXene increased, and more active sites are generated to improve its gas‐sensitive properties. After the last reaction time exceeds 8 h (Figure 3e) to 16 h (Figure 3f), MXene is completely coated with Cu‐HHTP. Moreover, the active sites generated by the growth on the interlayer and surface disappeared, causing a decrease in the area of accessible contact of MX@Cu.

It can be seen from in transmission electron microscope (TEM), the granular Cu‐HHTP marked by the small circle of dotted red lines surrounds the MXene marked by the pink dot plot as depicted in Figure 3g. The introduction of Cu‐HHTP retains the original 2D‐layered structure of MXene, and also increases the layer spacing of MXene, which can be observed from the wide gap between the deep black in Figure 3g. It is calculated that the bottom lattice spacing d ≈ 0.283 nm is the (200) plane of MXene, and the red circle of lattice spacing d ≈ 1.986 nm is the orifice of Cu‐HHTP, as shown in Figure 3h. Meanwhile, Figure 3i demonstrates that the two sides are combined, and the distance between Cu‐HHTP layers is d = 0.363 nm and is vertically stacked along the crystallographic c‐direction, exhibiting a spacing of 3.63 Å. Energy dispersive X‐ray spectroscopy (EDS) images of MX@Cu‐8 (Figure 3j) illustrate the uniform distribution of C, Ti, O, and Cu (Figure 3k–n), confirming the uniform growth of Cu‐HHTP nanoparticles throughout the MXene lamellar material and coating MXene.

**Figure 4:**
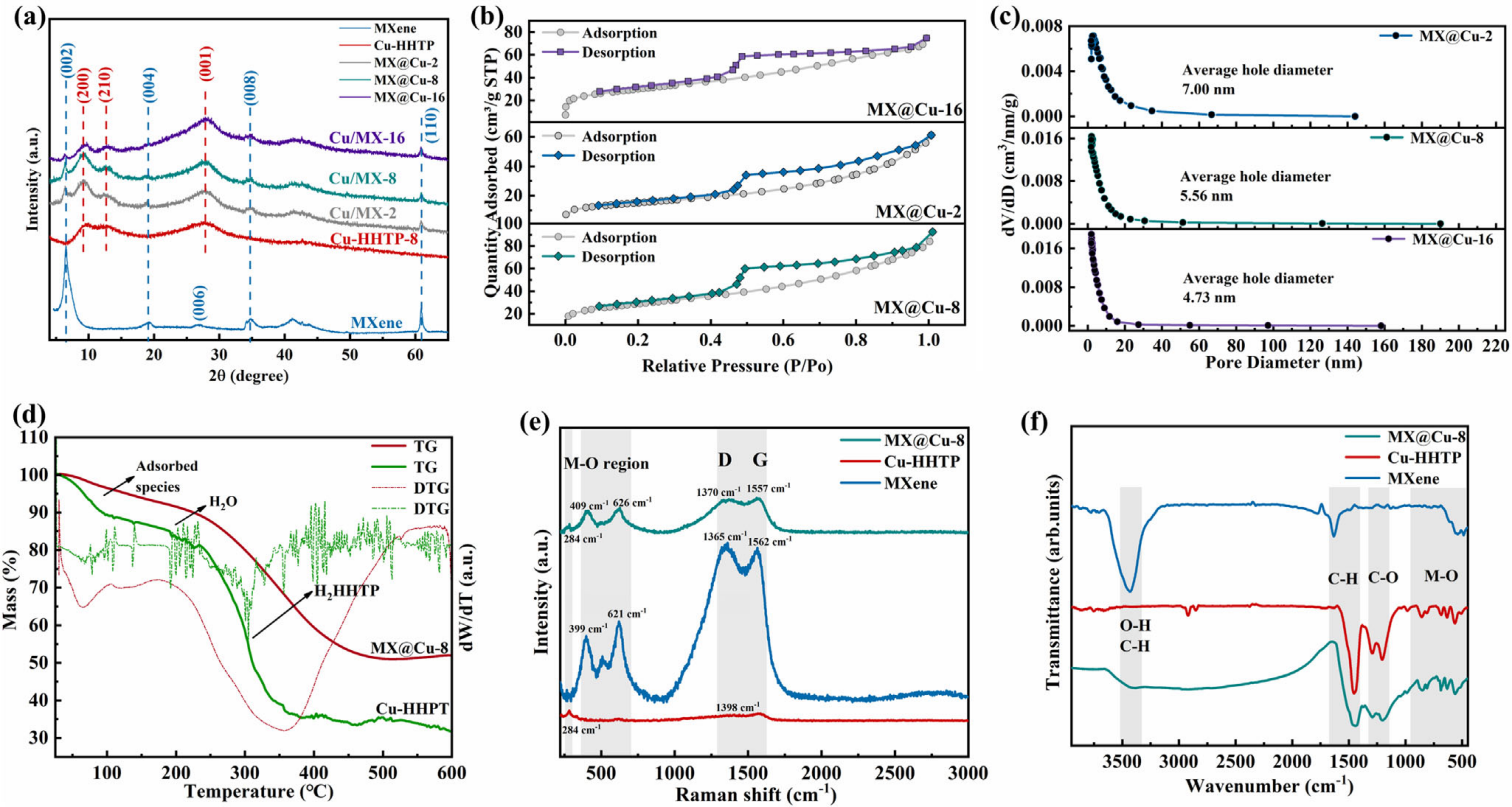
a) XRD patterns of MXene, Cu‐HHTP‐8, and MX@Cu‐T,b) N_2_ adsorption‐desorption isotherms, and c) Aperture of MX@Cu‐T, d) TG and DTG of MX@Cu‐8 and Cu‐HHTP‐8, e) Raman, f) FT‐IR.

As shown in the **Figure** **4**a, in the XRD pattern of MXene, the prominent (002) surface peak has a small angular deviation, indicating that the MXene distance treated by the organic intercalator becomes larger. Peaks of 2*θ* = 6.5°, 19.2°, 26.8°, and 34.8° in the figure correspond to (002), (004), (006), and (008), representing the synthesis of MXene,^[^ ^29^ ^]^ respectively. Few miscellaneous peaks with the smooth curve indicate that fewer layers of MXene material are successfully prepared.^[^ ^30^ ^]^ The XRD pattern of Cu‐HHTP shows peaks at 2*θ* values of 9.3°, 12.6°, and 27.8°. These peaks correspond to the (200), (210), and (001) reflections reported in the literature,^[^ ^31^ ^]^ which confirms the successful synthesis of Cu‐HHTP. By comparing the XRD of prepared MXene with that of prepared Cu‐HHTP‐8, the corresponding representative peaks are also found, and by comparing the peak ratio of MX@Cu with MXene (002), it can be discovered that the ratio decreases with the increase of time. Conversely, the peak ratio of MX@Cu to Cu‐HHTP (001) intensifies with the increase of time, indicating increase in the proportion of Cu‐HHTP in the composite with time. Therefore, XRD data exhibit the successful recombination of Cu‐HHTP and MXene.

The adsorption and desorption behavior of the sample MX@Cu‐T can be analyzed by the N_2_ adsorption and desorption isotherm test at 80 ℃ (Figure 4b). The N_2_ adsorption isotherms of the samples all increased sharply at lower relative pressures, in accordance with type IV isothermal adsorption line characteristics.^[^ ^32^ ^]^ As shown in Figure 4c, the distribution of narrow pores in the coordination framework is mainly concentrated ≈2 nm. The microporous structure of multilayer MXene remained intact after binding with Cu‐HHTP. The specific surface area of MX@Cu‐8 calculated by the BET method is 99.51 m^2^ g^−1^ and is nearly two times that of MX@Cu‐2, which is in accord with the above results of SEM and XRD. When the samples were reacted to 16 h, the BET of MX@Cu‐16 in Figure S3 (Supporting Information) was similar to that of MX@Cu‐8, and it can be seen that the pore size of MX@Cu‐16 is relatively smaller than that of MX@Cu‐8 in Figure 4b, which indicates that the MX@Cu‐16 has a relatively larger amount of MOF than MX@Cu‐8, and the effective channels provided by the MXene interlayers reduced or disappeared. The synergistic effect of porous MOF and multilayered MXene not only increases the specific surface area when the reaction reaches 8 h, but also exposes more active sites for the conducive establishment of more effective gas adsorption, enrichment and transport pathways.

In order to study the thermal stability, behavior of the prepared samples, thermogravimetric analysis (TGA), and differential thermogravimetric analysis (DTGA) of these samples are performed on Figure 4d. The overall pyrolytic weight loss trend of MX@Cu‐8 is shown to be similar to that of Cu‐HHTP‐8. And the overall stability of MX@Cu‐8 is improved relative to that of Cu‐HHTP‐8 due to the introduction of MXene. The first decay phase occurs before 80–130 ℃, and the gradual weight loss in this phase is attributed to be related to the evaporation of physiosorbed water and O_2_. A slight curvilinear decay occurs at 130–300 ℃, which is related to the vaporization of chemisorbed water. The weight loss in the final stage was attributed to the decomposition and collapse of the organic linker (H_2_HHTP) in the Cu‐HHTP framework. TGA analysis showed that the structure of the MX@Cu‐8 composites maintains good thermal stability until ≈220 ℃, a property that ensures sensing tests of the target gas at room temperature.

In Raman spectra (Figure 4e), the characteristic peaks of MXene (399 and 621 cm^−1^) and MX@Cu‐8 (409 and 626 cm^−1^) displayed at 150–1000 cm^−1^ are the metal‐oxygen bonding and metal‐bis (dioxolane) ring vibrational modes of MOF in the low‐energy vibrational mode. The C─H in‐plane bending mode of benzophenanthrene (1398 cm^−1^), as shown in Cu‐HHTP‐8 and MX@Cu‐8. The stretching of aromatic C─C bonds is evident from the characteristic Raman peaks, demonstrating that both MX@Cu‐8 (1370 and 1557 cm⁻¹) and MXene (1365 and 1562 cm⁻¹) exhibit D and G bands closely resembling those of graphene, a 2D carbon material.^[^ ^23^ ^]^

Fourier transform infrared spectroscopy (FTIR) also supports the successful insertion of Cu‐HHTP into MXene interlayers via spectral assignments based on vibrational modes, as shown in Figure 4f. Strong peaks at 1443 cm^−1^ are attributed to C─H bending.^[^ ^33^ ^]^ The strong peak at 1203 cm^−1^ is attributed to C─O stretching,^[^ ^34^ ^]^ and the peak < 1000 cm^−1^ corresponds to the M─O stretching mode.^[^ ^35^ ^]^

Electrochemical impedance spectroscopy (EIS) is a profound and specific analytical method to study the carrier transport properties of materials. Figure S4 (Supporting Information) shows the plots of Cu‐HHTP‐8, MXene, and MX@Cu‐8 composites with semicircular curves, which describe the electron transport resistance in the electrodes, reflecting the degree of diffusion of the transported carriers. The smaller EIS semicircle in the high‐frequency region indicates the lower charge transport resistance. It can be seen in Figure S4 (Supporting Information) that the radius of the MX@Cu‐8 composite material is relatively small compared to that of the Cu‐HHTP‐8, thus indicating that the MX@Cu‐8 material improves the carrier transport properties and favors the electron transfer. The introduction of MXene improves the transport properties of the carriers and increases the number of electrons transferred inside the material.

**Figure 5:**
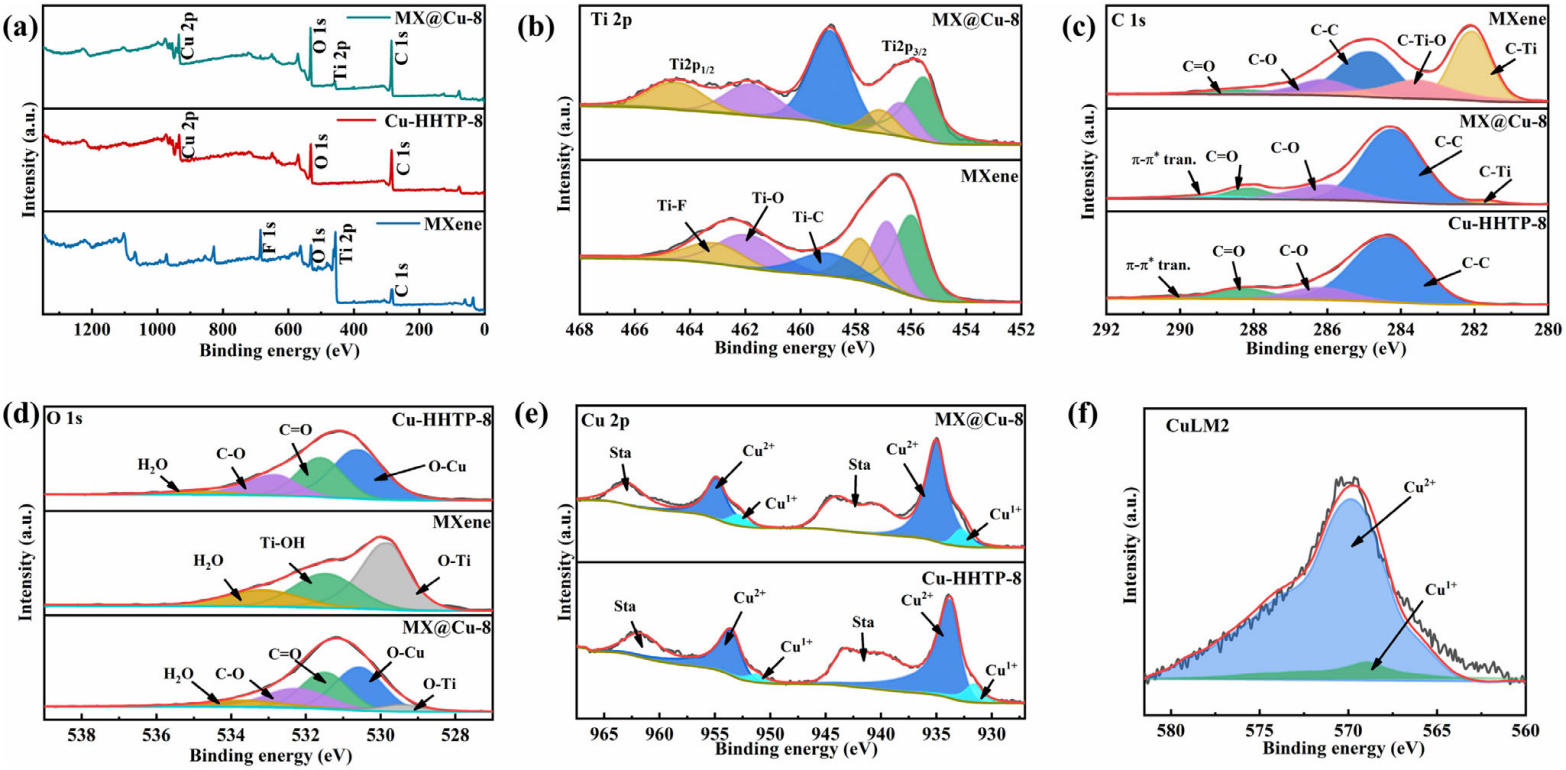
a) XPS survey spectrum, b) Ti 2p, c) C 1s, d) O 1s, and e) Cu 2p of MXene, Cu‐HHTP‐8, and MX@Cu‐8, f) Cu LM2 spectra.

The chemical composition and valence states of Cu‐HHTP‐8, MXene and MX@Cu‐8 composites are characterized and analyzed by X‐ray photoelectron spectroscopy (XPS). In **Figure** **5**a, the full XPS spectra presents the elements corresponding to Cu‐HHTP‐8, MXene and MX@Cu‐8 composites. The presence of Ti─C (459.1/456 eV) peaks in MXene, as seen in the Ti 2p maps (Figure 5b; Table S1, Supporting Information), indicating the successful synthesis of MXene.^[^ ^36^ ^]^ In addition, the presence of Ti─F (463.3/457.9 eV) and Ti─O (462.0/456.9 eV) peaks proves that the as‐prepared MXene contains abundant surface functional groups,^[^ ^37^ ^]^ including ─OH, ─F, and ─O. The existence of these oxygen‐containing functional groups can also be confirmed in the XPS plots of O 1s (Figure 5d). The peak area ratios and intensities of the Ti─F and Ti─O peaks in the MX@Cu‐8 composite have decreased compared to those of MXene in Figure 5b, exhibiting the copper ions adhered to the surface of MXene and occupied some of the surface functional groups to grow Cu‐HHTP and form the MX@Cu‐8 composite. In addition, a considerable difference between the C 1s and O 1s XPS peaks of Cu‐HHTP‐8 and MX@Cu‐8 composites further provides evidence for the growth of Cu‐HHTP on MXene. For Cu‐HHTP‐8, there are only four C 1s peaks at 289.3, 288.2, 286.3, and 284.4 eV, belonging to π–π*, C═O, C─O, and C─C bonds,^[^ ^32^, ^38^ ^]^ respectively (Figure 5c; Table S2, Supporting Information). In contrast, a C─Ti (282.1 eV) peak of MXene appeared in the MX@Cu‐8 composite except for the four typical characteristic peaks of Cu‐HHTP‐8. The presence of a C─Ti (282.1 eV) peak in MXene further reveal that the MXene material has been successfully synthesized and composited, and thus can be used as a nucleation site for MOF.

After the formation of Cu‐HHTP on MXene, the ratio of C─O and C═O peaks in the C 1s spectra increases due to the catecholate and semiquinone states of the HHTP ligand^[^ ^39^ ^]^ (Figure 5c). In the O 1s spectrum (Figure 5d), the O─Cu peak (530.6 eV) can be clearly deconvolved after Cu‐HHTP formation,^[^ ^40^ ^]^ which is a result of the coordination of the ligand with the metal center. The Cu 2p_3/2_ peak of MX@Cu‐8 (Figure 5e) exhibits an asymmetric shape for the redox activity of HHTP ligand capable of multiple oxidation states is present in the semiquinone and catecholate states, resulting in Cu^2+^ and Cu^+^ mixed valence metal,^[^ ^41^ ^]^ which can also be seen by Auger spectrograms of copper (Figure 5f). These properties theoretically support the strong sensitivity of Cu‐HHTP, which is described in the next section. In addition, the binding energy peaks of Ti 2p, C 1s, and O 1s in the MX@Cu‐8 composites are all shifted to lower binding energies compared to MXene, which is attributed to the fact that Cu‐HHTP is tightly assembled to the surface of MXene, which increases the electron density and forms an excellent composite.

### 2.2 Dynamic TEA Gas‐Sensing Characteristics

**Figure 6:**
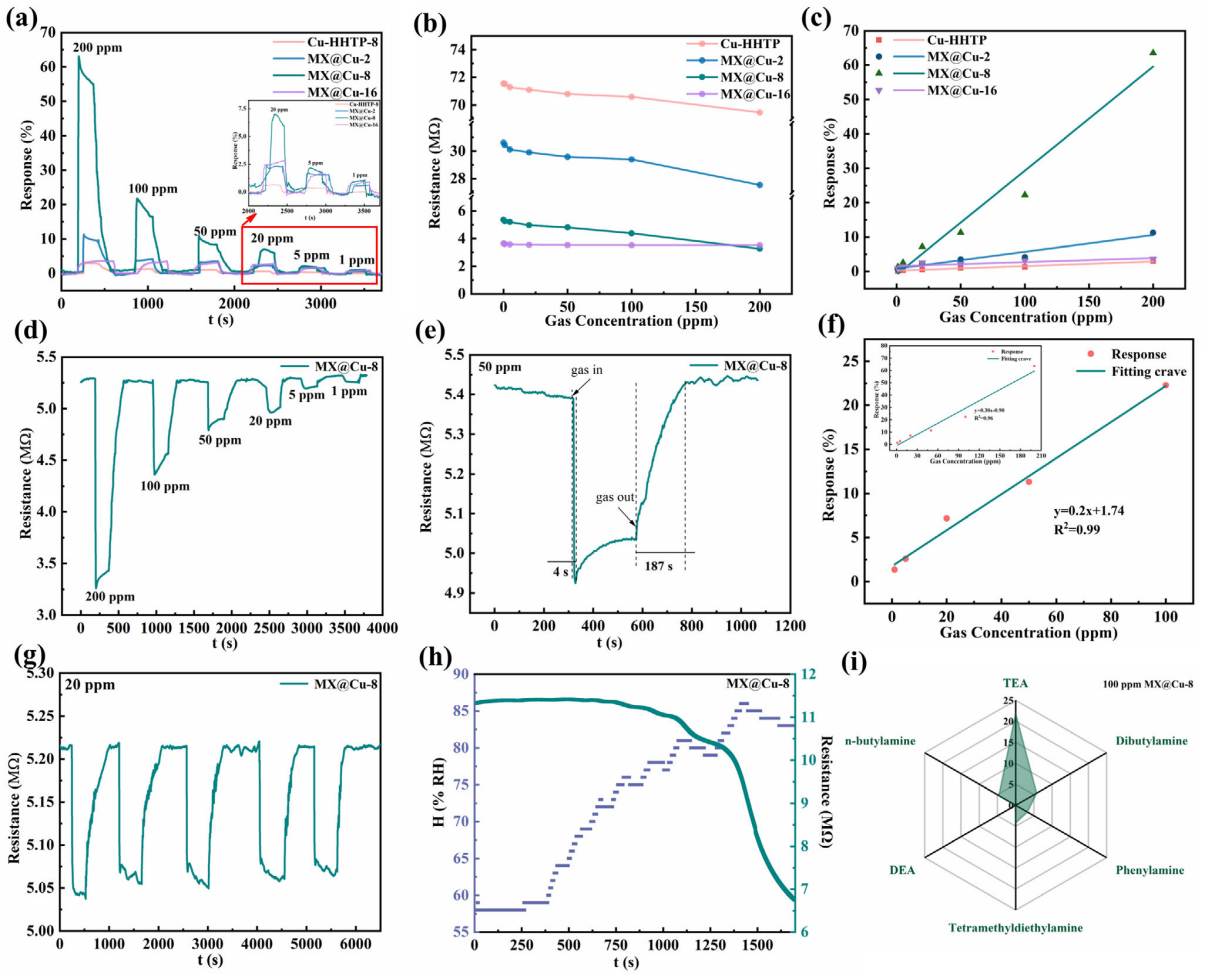
Gas sensing properties of the pristine Cu‐HHTP‐8 and MX@Cu‐T. a,b) Dynamical response/recovery curve and c) the linear relationship between the concentration and response value of Cu‐HHTP‐8 and MX@Cu‐T to different concentrations of TEA from 1 to 200 ppm at room temperature. d) The resistances for MX@Cu‐8 in the TEA concentration range of 1–200 ppm at room temperature. e) The duration required for the response and recovery of MX@Cu‐8 when exposed to 50 ppm TEA. f) The response value and concentration linear correlation of MX@Cu‐8 in a low concentration of TEA. g) Response/recovery times of the five repeated tests when exposed to 20 ppm TEA for MX@Cu‐8. h) Resistance to humidity. i) The response value (%) of MX@Cu‐8 toward various gases at a concentration of 100 ppm.

The device was operated below room temperature, and four sensors based on pristine Cu‐HHTP‐8 and MX@Cu‐T were tested for detailed gas response to different concentrations of TEA to investigate the best performance representation (**Figure** **6**a). Importantly, MX@Cu‐8 showed the highest gas response values regardless of the different concentrations, indicating that MX@Cu‐8 has the largest aspect ratio and the richest active sites in the range of these four samples. At room temperature, the gas response value of the MX@Cu‐8 sample to 200 ppm TEA reached 63.54% ≈21 times higher than that of the pure Cu‐HHTP‐8 (Figure 6a) for the gas‐sensitive performance assay of 200 ppm TEA.

In comparison, MX@Cu‐8 was significantly better than MX@Cu‐2 and MX@Cu‐16, with a greater degree of variation in resistivity values (Figure 6b; Figures S5–S7, Supporting Information). Therefore, the gas‐sensitive performance of MX@Cu‐8 was systematically tested in subsequent experiments. The response of the MX@Cu‐8 sensor to different concentrations (1–200 ppm) of TEA shows that the response value increases significantly with the increase of TEA concentration. Furthermore, the specific response values are 1.36%, 2.58%, 7.17%, 11.32%, 22.27%, and 63.54% corresponding to 1, 5, 20, 50, 100, and 200 ppm TEA, respectively.

The dynamic gas response curves of the Cu‐HHTP‐8 and MX@Cu‐T sensors at different TEA gas concentrations, the linear relationship between the TEA gas concentration and the response value is shown in Figure 6c and Figures S8–S10 (Supporting Information), respectively. The fitting equations of the MX@Cu‐2,8,16 sensor response to the concentration of TEA was Y_1_  =  0.05x  +  0.83, and the coefficient of determination (R^2^) was 0.98; Y_2_  =  0.30x – 0.90, with R^2^ of 0.96; and Y_3_  =  0.02x  +  1.31, with R^2^ of 0.77. In the low concentration range (1–100 ppm), MX@Cu‐8 had a better linearity R^2^ of 0.99, indicating a strong linear relationship between the response values in low concentration of TEA. The Cu‐HHTP‐8 sensor's dynamic gas response curves compared with the performance of the three groups of MX@Cu (Figures S8 and S9, Supporting Information), Y_0_  =  0.01x  +  0.28 at low concentrations, and the coefficient of determination (R^2^) was 0.89, indicating that the gas‐sensitive performance of MX@Cu was significantly better than that of Cu‐HHTP‐8 (Figure S10, Supporting Information).

The response‐recovery dynamic curves of Cu‐HHTP‐8 and MX@Cu samples toward 50 ppm TEA at room temperature are showed in Figure 6e and Figures S11–S13 (Supporting Information), respectively. MX@Cu‐8 has a response time of 4 s and a recovery time of 187 s, which is superior to that of Cu‐HHTP‐8 (τ_res_ = 16 s, τ_rec_ = 76 s), MX@Cu‐2 (τ_res_ = 19 s, τ_rec_ = 69 s), and MX@Cu‐16 (τ_res_ = 123 s, τ_rec_ = 28 s). Good reproducibility is an important criterion for evaluating the sensing performance. Figure 6g and Figures S14–S16 (Supporting Information) demonstrated the cyclic testing of MX@Cu and Cu‐HHTP‐8 nano gas‐sensitive materials exposed to 20 ppm TEA for five consecutive times, showing that the response values of the MX@Cu gas‐sensitive sensors were almost the same at the same TEA concentration. The humidity tolerance of the MX@Cu‐8 sensor was evaluated over time (Figure 6h). The results demonstrated that under humidity levels below 80%, the sensor maintained stable performance with minimal fluctuation. On the contrary, when the humidity was 80%, the resistance dropped rapidly and fluctuated greatly.

In addition, selectivity is a critical parameter for sensors to be viable for practical applications. When the gas conditions in the environment where the sensor is placed are complex, it is relevant for the sensor to be able to respond accurately and efficiently to the information of the target gas. Figure 6i demonstrates the selective gas sensitivity test diagram of MX@Cu‐8 sensor. In order to investigate the selectivity of MX@Cu‐8 sensor for TEA, the measurement of the gas at room temperature against 100 ppm of five other organic amine interfering gases (dibutylamine, phenylamine, tetramethyldiethylamine, diethylamine (DEA), n‐butylamine) and volatile organic compounds (VOCs) have been conducted to show a super gas‐sensitive capability. Moreover, the sensor's selectivity toward common VOCs, including ammonia (NH_3_), ethanol (EtOH), acetone (ACE), dimethylformamide (DMF), and methanol (MeOH) was investigated. From Figure 6i and Figure S17 (Supporting Information), it can be seen that the prepared sensors have a higher response to TEA than to the other ten interfering gases, manifesting significant gas selectivity, and can realize complete response to triethylamine under a complex gas environment.

**Figure 7:**
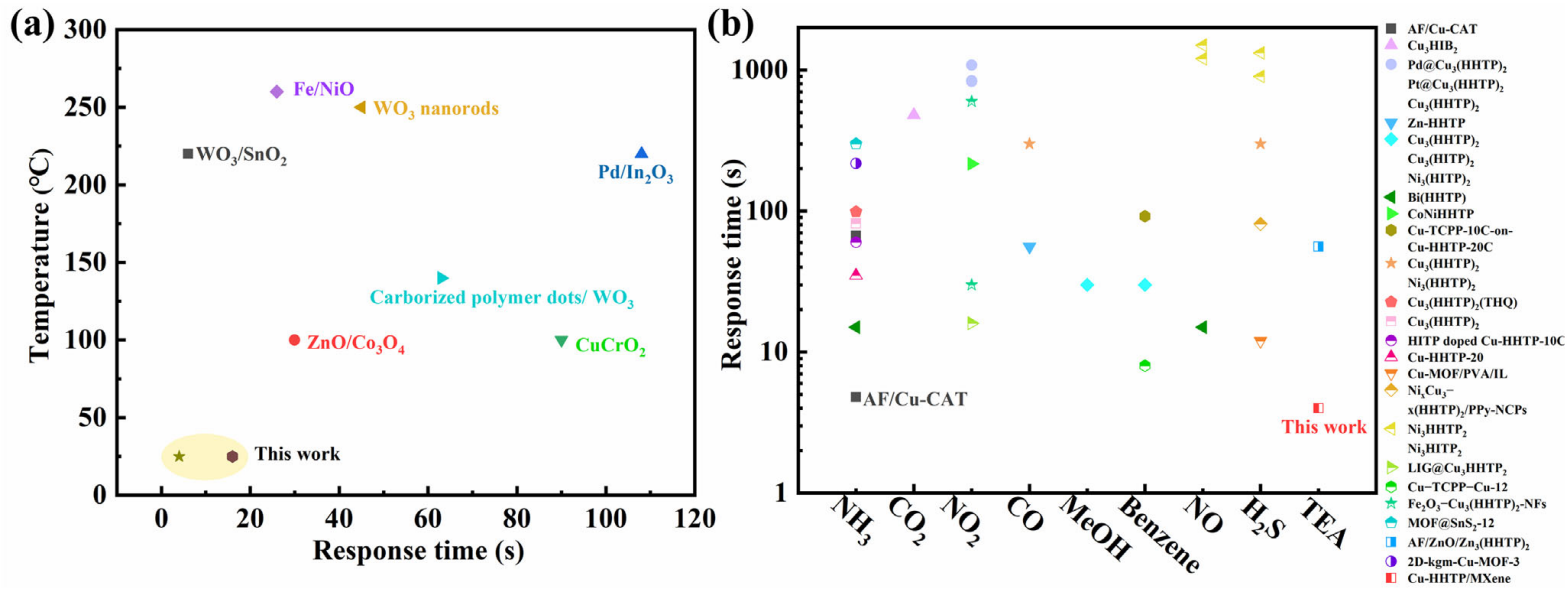
MX@Cu‐8 compared with (a) metal oxides and (b) conductive MOFs.

Compared with the generally higher operating temperatures of conventional metal oxides, MX@Cu composites can detect TEA at room temperature and concentrations below 1 ppm. The comparison is shown in the following **Figure** **7**a and Table S3 (Supporting Information). There are relatively few researches on the use of conductive MOFs in TEA detection, which fills the gap in this research field, and has superior performance in response recovery time compared with the most cutting‐edge conductive MOFs (Figure 7b; Table S4, Supporting Information).

Characterization analyses, including SEM and BET, indicate that the MX@Cu‐16 material exhibits a denser structure, leading to a significantly prolonged response time (τ_res_ = 123 s) compared to MX@Cu‐8 (τ_res_ = 4 s). This delay is primarily due to the dense architecture of MX@Cu‐16, which hinders the diffusion of gas molecules within the material, resulting in slower response and recovery rates as the molecules navigate through confined nanochannels. In contrast, MX@Cu‐8 integrates microporous MOFs with MXene layers featuring wider interlayer spacing, which not only enhances the specific surface area but also promotes efficient mass transfer of gas molecules to active sites (such as open metal sites or ligands) within the Cu‐HHTP micropores. Moreover, the inherent advantages of MOFs, including their abundant open metal centers and functional edge ligands capable of interacting with gas molecules, are fully leveraged. These mechanisms collectively explain why MX@Cu‐8 demonstrates a significantly faster response time at room temperature.^[^ ^42^ ^]^

### 2.3 Mechanisms of TEA Sensing

**Figure 8:**
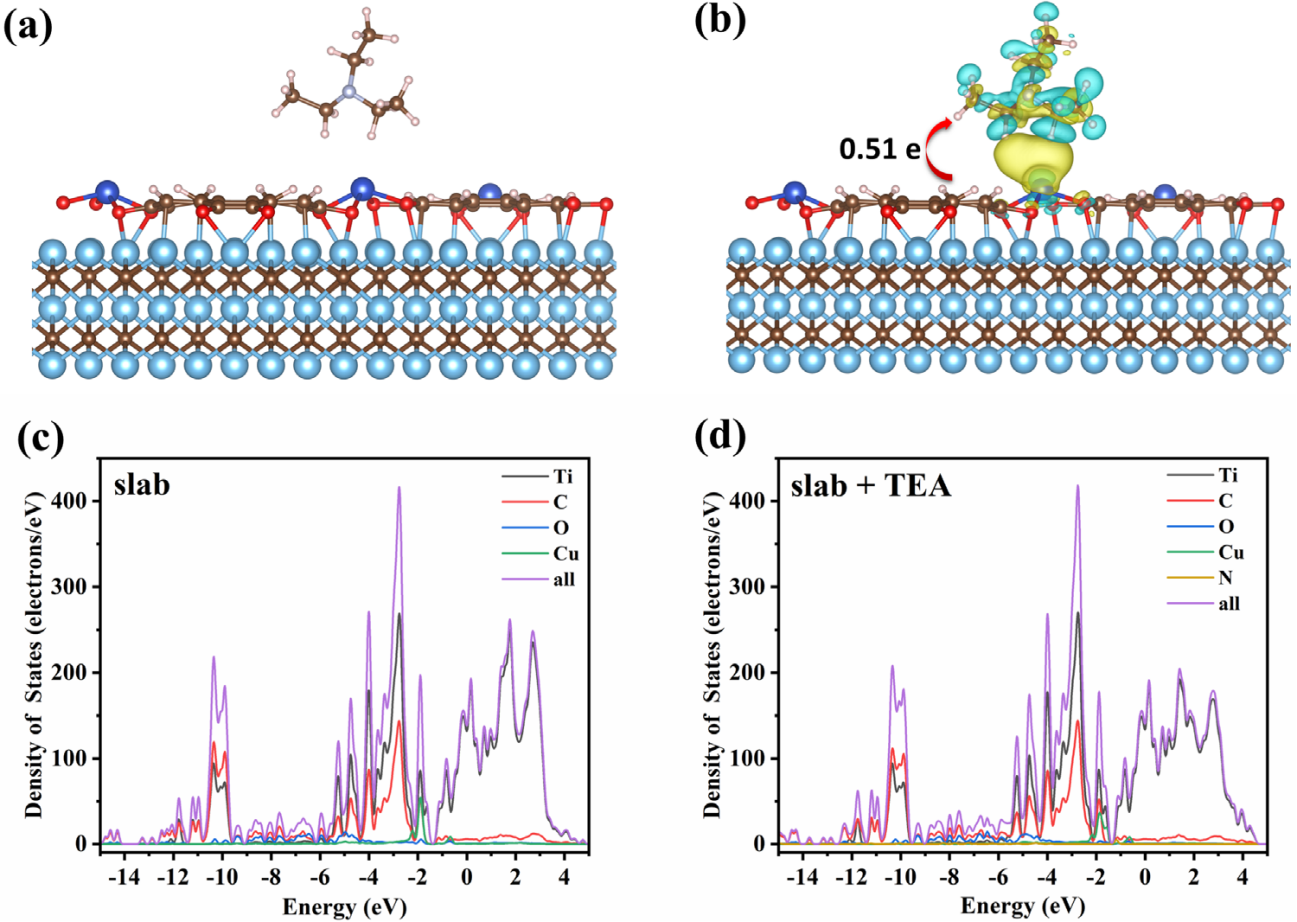
a) Cu adsorption sites of Cu‐HHTP in MX@Cu composite. b) The differential charge density distribution diagram in the MX@Cu/TEA systems. c,d) PDOS of C, O, Cu, Ti atoms and total curves in MX@Cu and MX@Cu/TEA systems.

Designing a sandwich structure aims to both enhance the TEA adsorption and amplify the electron signal transition, which then contributes to improving the sensor performance. To clarify the sensing mechanism, density functional theory (DFT) calculations were conducted. As shown in Figure 6a, Cu‐HHTP nanoparticles in the MX@Cu sensor predominantly drive TEA adsorption. Among the potential adsorption sites (Cu, C, O), TEA molecules spontaneously adsorb onto Cu atoms, forming the most stable configuration (**Figure** **8**a). The calculated adsorption energies (TEA‐Cu < TEA‐C < TEA‐O, shown in Figure S18, Supporting Information) indicate stronger spontaneity for TEA‐Cu, confirming the spontaneity and selectivity of this interaction. Density of states (DOS) analysis (Figure 8c,d) reveals negligible electronic structure changes in MX@Cu after TEA adsorption, indicating preserved structural stability. Differential charge density analysis (Figure 8b) demonstrates electron transfer from Cu atoms (depletion, blue regions) to TEA's N and O atoms (accumulation, yellow regions), highlighting the charge transfer mechanism. These results collectively explain the enhanced sensing performance through stable adsorption and efficient electron transfer without structural degradation.

**Figure 9:**
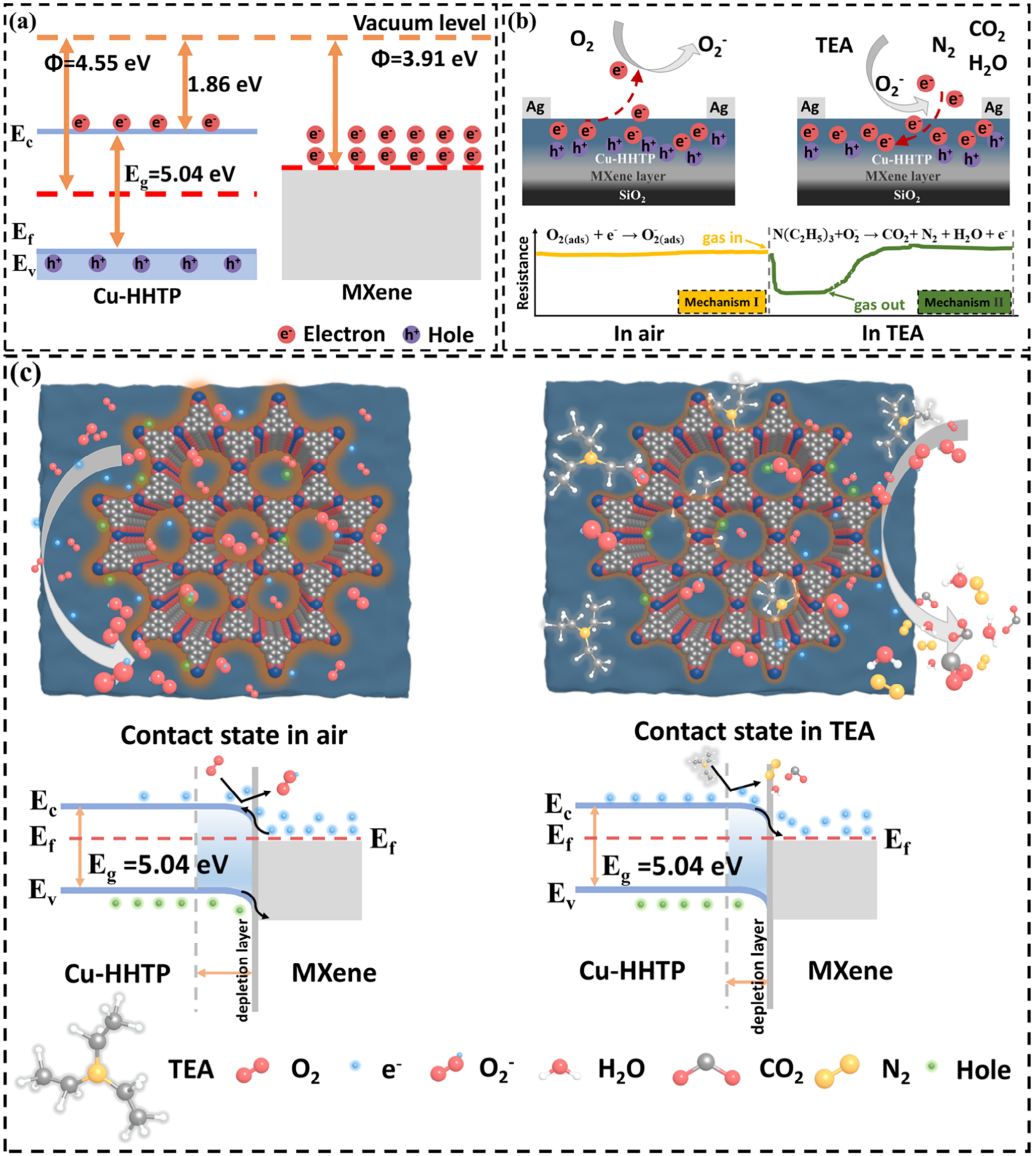
a) The schematic energy band diagrams of Cu‐HHTP and MXene in a separate state. b) Schematic diagram of charge transport on a material surface. c) 3D sensing mechanism diagrams of heterostructured MX@Cu and corresponding depletion model and band diagram are represented.

There is no doubt that the MX@Cu gas sensor shows ideal gas‐sensitive characteristics. Moreover, the high sensitivity should be related to two kinds of interactions: 1) the interaction between the analyte and the large conjugated unit, and 2) the strong metal‐analyte interaction. Among them, the amplification effect of the heterojunction formed in situ between Cu‐HHTP‐8 and MXene plays a crucial role in improving the performance of gas sensors. In addition, the Fermi level (E_F_) and the maximum valence band level (E_V_) of Cu‐HHTP and MX@Cu‐8 with respect to the vacuum level (E_VAC_) were determined by UPS spectral analysis (**Table** **1**; Figures S19 and S20, Supporting Information). In order for photoelectrons to escape from the surface of the sample, sufficient energy is required; the incident photon energy is equal to the sum of the binding energy (relative to E_F_) and the work function (Φ),^[^ ^25^ ^]^ where Φ = E_VAC –_ E_F_. As shown in Figures S19 and S20 (Supporting Information), the line on the left represents the secondary electron cutoff edge (high binding energy edge) with an energy of ≈16.67 eV, determined by the intersection of the linear part of the spectrum and the baseline. For a fixed incident photon energy (hv = 21.22 eV), the work function of this sample is calculated as Φ = 21.22–16.67 = 4.55 eV, so the E_F_ relative to E_VAC_ is determined as −4.55 eV. Based on the Fermi level (low binding energy edge, determined by the linear part of the spectrum intersecting with the baseline), the difference between E_F_ and E_V_ is 2.35 eV. Thus, E_V_ is determined to be −6.90 eV relative to E_VAC_. Then, the bandgap (*E*_g_) of Cu‐HHTP‐8, MXene, and MX@Cu‐8 were estimated by UV–vis (Figure S21, Supporting Information) fitting to 5.04, 4.16, and 4.63 eV, respectively. From the latest reports, we know that MXene (Φ = 3.91 eV) is like a metal conductor,^[^ ^43^ ^]^ and the energy band separates the state with Cu‐HHTP, as shown in **Figure** **9**a.

**Table 1:** Band energy of Cu‐HHTP and MX@Cu.

Materials	*E*_F_ [eV]	Φ [eV]	*E*_F_‐*E*_v_ [eV]	*E*_v_ [eV]	*E*_g_ [eV]	*E*_c_ [eV]
Cu‐HHTP	16.67	4.55	2.35	−6.90	5.04	−1.86
MX@Cu	17.08	4.14	3.66	−7.80	4.63	−3.17

During the gas sensitive performance test, as shown in Figure 9b, the resistance of the sensor based on MX@Cu composites decreased when detecting TEA. 2D MXene sheet has metallic properties owing to the MXene work function (φ_MXene_) is smaller than the Cu‐HHTP work function (φ_Cu‐HHTP_), the band of Cu‐HHTP will be bent until the Fermi level equilibrium, at which time a thick electron depletion layer will be produced. Figure 9c illustrates a 2D MXene sheet and P‐type Cu‐HHTP nanoparticles forming Schottky heterojunction.^[^ ^44^ ^]^ The charge‐depletion layer formed by the Schottky heterojunction obstructs the movement of electrons and increases the resistance of the composite. When TEA molecules are adsorbed on the surface of MX@Cu composites, the TEA molecules inject electrons into the surface of Cu‐HHTP. This process increased the Fermi level of Cu‐HHTP, narrowed the gap between the MXene and Cu‐HHTP work function, and reduced the width of the space charge region, thus improving the electron transport efficiency and reducing the resistance.

In addition to the formed MX@Cu heterojunction, the highly oriented porous structure of the Cu‐HHTP layer also promotes the gas‐sensitive properties, and experiments have further demonstrated that the [001] oriented large conjugated unit of Cu‐HHTP electron transfer is faster because the electron transfer rate on the c‐axis is higher than that on the ab‐plane. When MX@Cu is exposed to air at room temperature, oxygen molecules tend to adsorb on the surface of Cu‐HHTP layer and capture free electrons in the conduction band, resulting in chemisorbed oxygen species^[^ ^1^ ^]^ (O_2(ads)_).

The charge transfer on the silver finger fork electrode was shown in Figure 9b. This process caused the band to bend upward, and produces an electron‐absorbing region on the surface of Cu‐HHTP, resulting in the formation of an electron depletion region and forming a high sensor resistance (Figure 9b mechanism Ι). In this work, all gas sensitivity tests were performed at room temperature and 3D sensing mechanism^[^ ^45^ ^]^ diagrams of heterostructured MX@Cu are displayed in Figure 9c. Thus, the adsorbed oxygen species can be described as follows (Equations (1), (2)):

(1) $$O_{2\left(\mathrm{gas}\right)}\to O_{2\left(\mathrm{ads}\right)}$$

(2)

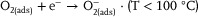




When the sensor is exposed to TEA, the TEA molecules will be oxidized by O_2(ads)_, releasing electrons back into the Cu‐HHTP and lowering the electron depletion layer and voltage barrier height,^[^ ^25^ ^]^ leading to a reduced resistance of the sensor to the gas sensing signal (Equation 3).

(3) $$N{\left(C_{2}H_{5}\right)}_{3}+O_{2}^{-}\to CO_{2}+N_{2}+H_{2}O+e^{-}$$

The excellent sensing performance of MX@Cu sensors is attributed to the porous nanostructure, including a suitable particle size and abundant in‐plane mesoporous. First, the MX@Cu prepared by the template method grows through the nucleation site provided by MXene, which increases the specific surface area and the active site and reduces the aggregation of Cu‐HHTP. The support of Cu‐HHTP prevents MXene from self‐stacking, greatly shortens the transport path, and facilitates the adsorption of gas molecules on the material surface. The gas‐sensitive response curve of MXene toward TEA (Figure S22, Supporting Information) reveals that the material exhibits virtually no detectable response to this analyte. Meanwhile, the DFT calculations confirm that in the MX@Cu sensing system, MXene primarily enhances electron transfer efficiency, whereas Cu‐HHTP functions as the active component for gas detection. EIS results quantitatively demonstrate this functional division: MXene exhibits the lowest impedance with optimal electron transfer capability, while pristine Cu‐HHTP shows the highest impedance. The integration of MXene into MX@Cu effectively accelerates electron migration kinetics, enabling synergistic enhancement in TEA sensing performance through complementary contributions from both components. The gas‐sensitive response to TEA was effectively improved. In conclusion, an efficient sensor for TEA gas detection based on MX@Cu has been successfully prepared.

**Figure 10:**
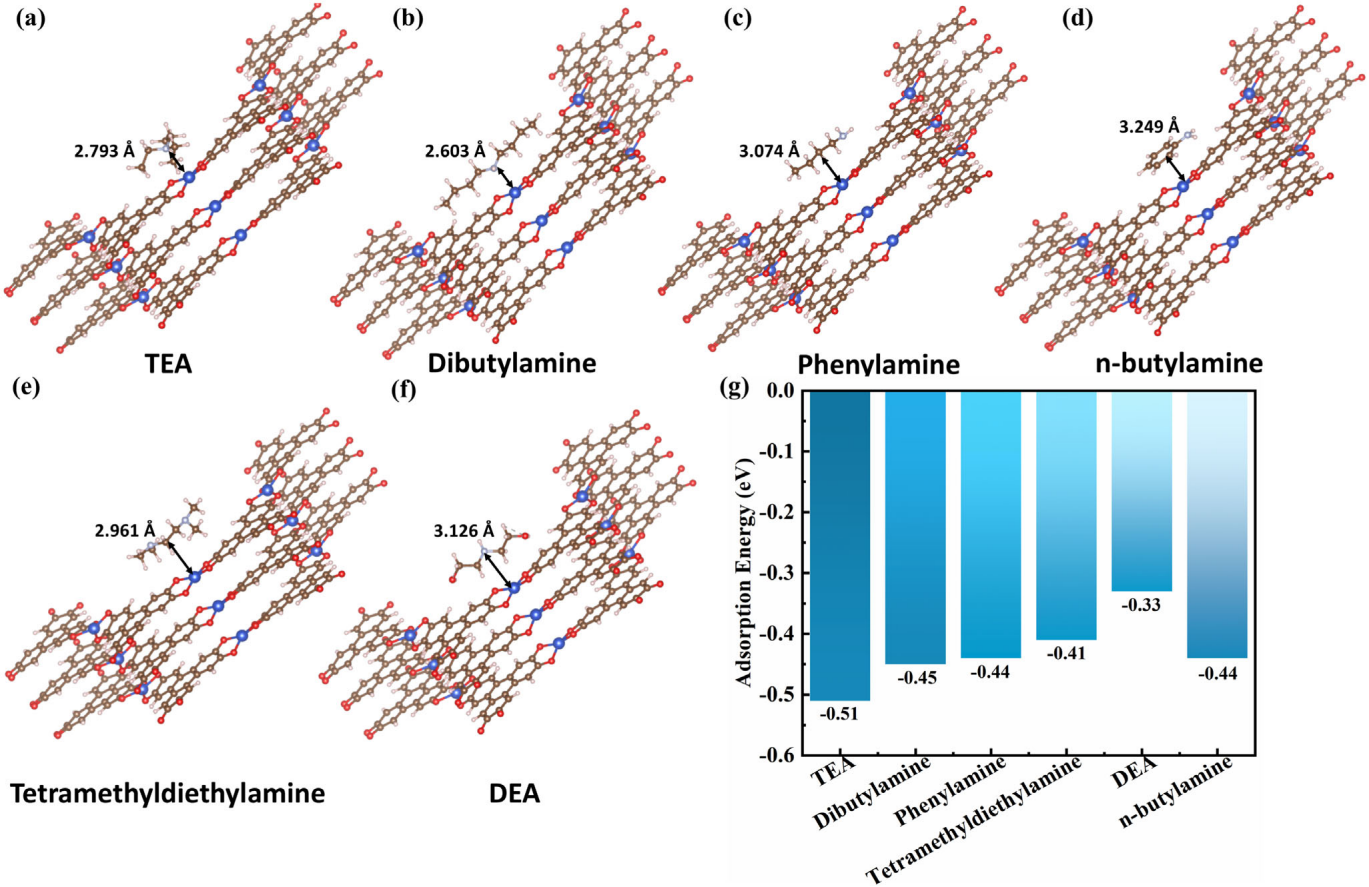
a) The optimized structures after the adsorption of TEA, b) dibutylamine, c) phenylamine, d) n‐butylamine, e) tetramethyldiethylamine, f) DEA over Cu‐HHTP, respectively. g) Histogram of adsorption energy of different gas molecules on Cu‐HHTP.

To investigate the adsorption properties of MX@Cu concerning 6 organic amine gases, we employed a DFT‐based computational approach to simulate the adsorption processes of TEA, dibutylamine, phenylamine, n‐butylamine, tetramethyldiethylamine, and DEA on the surface of Cu‐HHTP. As shown in **Figure** **10**, the inherent porous structure of Cu‐HHTP revealed numerous active edge sites, facilitating the enhancement of gas adsorption. Notably, the adsorption configuration of TEA molecules on Cu‐HHTP, the bond length of the adsorption bond is 2.793 Å, the adsorption energy is −0.51 eV, and the TEA gas molecules can form effective adsorption on the surface of Cu‐HHTP. The comparative absolute adsorption energies indicated the following order of adsorption strength: TEA (−0.51 eV) > dibutylamine (−0.45 eV) > phenylamine (−0.44 eV) = n‐butylamine (−0.44 eV) > tetramethyldiethylamine (−0.41 eV) > DEA (−0.33 eV), underscoring the superior adsorption performance of TEA on the Cu‐HHTP surface (Figure 10g). Furthermore, the integration of MXene significantly enhances electron transport capacity, suggesting that the MX@Cu heterostructure exhibits remarkable selectivity and sensitivity toward TEA gas.

### 2.4 Wireless Sensors Detect Food Freshness

**Figure 11:**
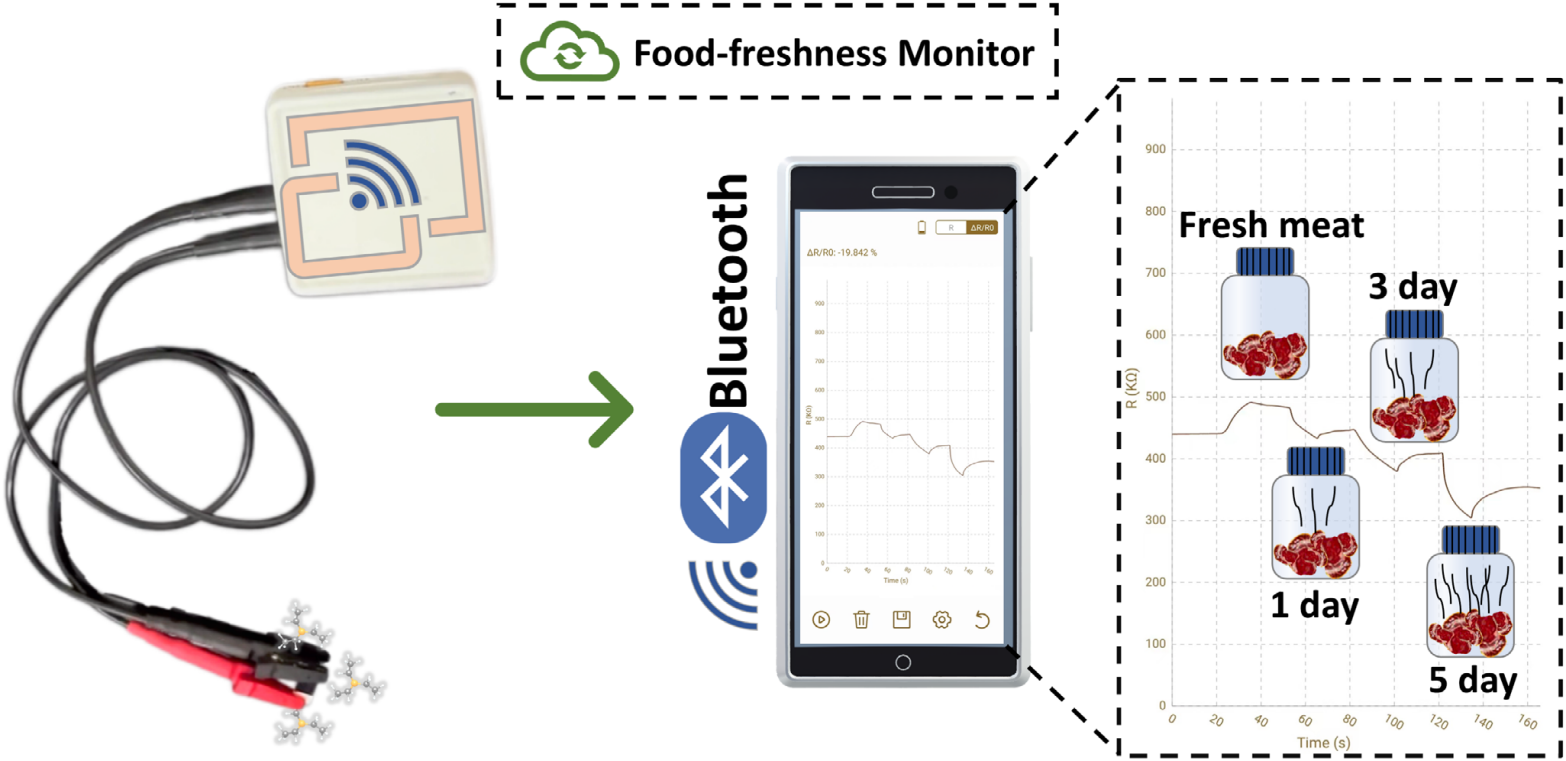
Schematic diagram of meat freshness detection using an MX@Cu‐based portable device.

The portable food freshness detection function, as an integral part of daily life use, is one of the future applications of this type of sensor. The portability and wireless connectivity of food freshness sensors are also very useful in the food market and other areas. Therefore, we integrated the MX@Cu sensor with the Bluetooth module for wireless data transmission, through which the output signal is transmitted to the mobile phone, to obtain a fully cloud‐based food freshness monitoring device (**Figure** **11**). Video S1 (Supporting Information) shows the function of the device, with the horizontal and vertical coordinates of the resistance (KΩ) and test time (s) of the MX@Cu sensor, respectively. MX@Cu sensors were used to monitor the freshness of raw pork and assess the impact of changes over time on the degree of spoilage. During pork spoilage, amine gases^[^ ^46^ ^]^ constitute the primary gaseous indicators, whereby the sensor can be used for detection. Frequently, the concentration of spoilage gas in meat containers gradually increases as meat rots. The change of sensitivity within a certain time is related to the degree of meat spoilage. Here, we examined changes in sensitivity over 30 s to assess the degree of meat spoilage. To standardize the testing process, four servings of 100 g fresh pork stored in bottles with a diameter of 10 cm (to avoid any effects of packaging) were prepared, and marked number 1 in the refrigerator and freeze at −18℃, number 2, 3, and 4 at room temperature for 1, 3 and 5 days, respectively. The MX@Cu sensor is also placed in a glass bottle for testing spoilage gases with pork (as exhibited in Video S1, Supporting Information). The same MX@Cu gas sensors have been employed to continuously conduct four tests at the same experimental conditions. Obviously, as storage time increases, meat corrupts much faster. Through the test phone, the relevant gas sensing tests for fresh meat removed from the refrigerator showed a slight increase in resistance. This occurs due to alcohol production in frozen pork^[^ ^47^ ^]^ coupled with the sensor's ascending response curve to alcohol concentrations (Figure S23, Supporting Information). The relative response speed of meat 3 and 4 is faster, and the resistance value changes more significantly (Figure S24, Supporting Information). We can easily judge the degree of meat spoilage through the resistance change of the sensing signal for rapid detection and continuous monitoring, which cannot be easily achieved by traditional detection methods. This lightweight and small size promotes its integration into any device that requires real‐time monitoring, and the entire process of food status can be monitored and even recorded in real time, ensuring food safety.

## 3 Conclusion

In conclusion, 2D‐cMOF Cu‐HHTP has been synthesized by assembling a catechol framework on the MXene layer to form MX@Cu. The support of MXene makes full use of the advantages of MOF's large surface area and porosity, increasing the surface‐active site and deepening the reaction degree, and achieving rapid sensing at room temperature without external energy (light or heat source). MX@Cu‐based gas sensors have the merit of high response (≈63.54%) to 200 ppm TEA, moisture resistance (RH < 80%), and short response and recovery time (4 s/187 s). The fast response and resilience of 4 s is far superior to most catechol skeleton sensors. The sensor can monitor TEA less than 1 ppm, meeting the detection requirements of NIOSH and ACGIH standards, responding quickly in the daily environment, and warning people to react in a short time. Besides, the freshness of meat is actually monitored with a portable device, and the signal is transmitted via a Bluetooth link to a wireless smartphone application to display real‐time resistance changes. This work not only makes the sensor's monitoring in the daily complex environment an important step forward, but also provides a new idea for the application of low‐power devices.

## 4 Experimental Section

### Materials

2,3,6,7,10,11‐Hexahydrotriphenylene hydrate (HHTP, 95.0%), dimethyl sulfoxide (DMSO, 99.99%), Lithium fluoride (LiF, 99.99%), and copper (II) acetate monohydrate (Cu(OAc)_2_·H_2_O, 99.5%) were purchased from Macklin. Ti_2_AlC_3_ (99.9%) was purchased from Beijing Beike New Material Technology Co., Ltd. Hydrochloric acid (HCl, 36–38 wt%) was purchased from Sinopharm Chemical Reagent Co., Ltd. Triethylamine (TEA, 99.0%) was purchased from BeiJing ShiJi. Ethanol absolute (EtOH, 99.7%) was purchased from BeiJing Tong Guang Fine Chemicals Co., Ltd. All the chemical reagents were used as received without further purification.

*Synthesis of Cu‐HHTP*

A 50 mg Cu(OAc)_2_·H_2_O (0.935 mmol) and 35 mg HHTP (0.454 mmol) were added to 35 mL absolute ethanol and ultra sounded for 15 min to form a dark brown solution. The mixed solution was then put into a 40 mL glass vessel and transferred to a blast drying oven at 45℃ for 8 h. The centrifuged deposit was washed with deionized (DI) water and EtOH 3 times, respectively. Finally, the material was subjected to a vacuum‐drying procedure at 60℃ for 24 h. A Schematic of the Cu‐HHTP synthesis steps is illustrated in Figure 1.

### Synthesis of Ti_3_C_2_T_X_ MXene Sheets

Dispersed multilayer MXene nanosheets were obtained through the process of in‐situ HF etching.^[^ ^48^ ^]^ Generally, 1.0 g LiF was placed in a 100 mL‐Teflon beaker and then 20 mL 9 m hydrochloric acid was added under magnetically stirred (700 rpm) in a fume hood. After the complete dissolution of 1.0 g Ti_3_AlC_2_, power was carefully added 5 times to avoid initial overheating of the solution. The mixture was then heated and stirred in a constant temperature water bath at 40 ℃ for 24 h. The result solution was poured into a centrifuge tube and centrifuged at 8000 rpm for 5 min. After washing with 2 M HCl 3 times, washing and centrifuging with DI water until the pH of the supernatant is ≈6, the process is repeated 8 times. The sediment was then collected and dried in a vacuum drying oven for 12 h to obtain multiple layers of Ti_3_C_2_T_X_ MXene. Few layers of Ti_3_C_2_T_X_ MXene can be further obtained by dispersing multiple layers of powder into DMSO.^[^ ^49^ ^]^

### Synthesis of MXene@Cu‐HHTP

A 10 mg few‐layer Ti_3_C_2_T_X_ MXene sheets prepared above were dispersed into 10 mL ethanol solution containing 0.935 mmol Cu(OAc)_2_·H_2_O. Subsequently, the mixed solution was subsequently poured into 25 mL ethanol solution containing 0.454 mmol HHTP. After ultrasonic for 15 min, the solution was separated evenly into three 40 mL vessels and transferred to a blast drying oven at 45 °C for durations of 2, 8, and 16 h, respectively. Treatment and collection of the final precipitate were the same as those of Cu‐HHTP. The Ti_3_C_2_T_X_ MXene@Cu‐HHTP composite was named as MX@Cu‐T (Cu, MX and T signifying Cu‐HHTP, MXene and the reaction time, respectively). A Schematic of the MX@Cu‐T synthesis steps is illustrated in Figure 2.

### Characterization

The crystal structure was ascertained through the implementation of an X‐ray diffractometer (XRD, Bruker D8 ADVANCE diffractometer) using Cu Kα radiation (λ = 0.154 nm). The morphology characteristics and element distributions of pristine Cu‐HHTP, MXene and MX@Cu‐T composites were meticulously investigated by scanning electron microscopy (SEM, SU8010). The 3H‐2000PS2 sorption analyzer was utilized to specific surface area (BET) and pore size. A micro‐Raman spectrometer equipped with a 532 nm laser was employed for the acquisition of Raman spectra. The surface chemical state was analyzed using X‐ray photoelectron spectroscopy (XPS).

### Gas Sensing Measurements

The gas‐sensitive analysis test system (CGS‐MT, Zhongju Tech, China) was employed to discern specific gases and gather the relevant data. The procedural steps for the fabrication of the triethylamine gas‐sensitive detection device are outlined as follows. Initially, the prepared sample (10 mg) is combined with ethanol (1 mL) in an agate mortar and meticulously ground until a paste is formed. Subsequently, the derived paste sample is uniformly applied onto the Ag‐Pd electrode sheet measuring 14×7 mm^2^. Following this application, the triethylamine gas sensor is meticulously obtained after allowing it to air‐dry at ambient temperature for a period of 24 h. Finally, the gas sensor undergoes a continuous aging process at 100 ℃ for 12 h, ensuring the attainment of a steadfast resistance in ambient air conditions. During the experiment, a quantity of triethylamine solution (99.0%) is introduced into the evaporator and heated at 100 ℃. Subsequently, the resulting solution is homogenized using the built‐in fan, thus generating a homogeneous gas environment. The concentration of triethylamine gas is determined utilizing the formula previously elucidated in the prior publication (Equation 4).

(4) $$Q=\frac{V\times C\times M}{22.4\times d\times \rho }\times 10^{-9}\times \left(273+T_{g}\right)/273+T_{B}$$

Here, the variables are defined as follows:

Q: volumetric quantity of the injected liquid (mL);

V: volume of gas chamber (L);

M: relative molecular mass of the substance under consideration;

ρ: purity of injected liquid;

C: concentration of prepared gas (ppm);

d: corresponds to the density of the injected liquid (g/cm^3^);

T_g_: test temperature (℃);

T_B_: temperature of gas chamber (℃).

According to static test mode, recorded the resistance change of MX@Cu‐T gas sensor by CGS‐MT, and response is defined as S =|R − R_0_|/R_0_ × 100%, where R and R_0_ represent initial resistance and reaction resistance, respectively. The response time is ascertained as the time when the response reaches 90% of the equilibrium value subsequent to the introduction of triethylamine gas, and the recovery time is determined as the duration it takes for the original response to reach 10% upon the return of triethylamine to the ambient air.

## Conflict of Interest

The authors declare no conflict of interest.

## Supporting information



Supporting Information

Supplemental Video 1

## Data Availability

The data that support the findings of this study are available from the corresponding author upon reasonable request.
