# Supplementary material for: 2D Conductive MOFs Intercalated in MXene Interlayer for Fast and Trace Detection of Triethylamine at Room Temperature
Source: Adv Sci (Weinh). 2025 May 24;12(25):2500786. doi: 10.1002/advs.202500786 (PMC12224948; doi:10.1002/advs.202500786)
Supplement: Supplementary file 1 — Supporting Information [file ADVS-12-2500786-s002.docx]

Supporting Information

Two-dimensional conductive MOFs intercalated in MXene interlayer for fast and trace detection of triethylamine at room temperature

Hao Zhang^a, b^§, Wei Cao^a^§, Jingfeng Wang^a^, Lei Guo^a^, Pu-Hong Wang^a^, Zhi-jun Ding^a^^[[1]](#footnote-1)^*, Lingmin Yu^b^*

^a^ State Key Laboratory of NBC Protection for Civilian, 102205, China

^b^ Xi’an Technological University, School of Materials and Chemical Engineering, Xi’an, Shaanxi, 710021, China

§ These authors contributed equally to this work.

The gas sensing assessment was conducted using the CGS-MT gas sensing test system to evaluate the material's moisture resistance through a controlled water vapor exposure process. In this process, DI water is evaporated via the device's heating stage, and the humidity within the test chamber is accurately monitored using a hygrometer. The experimental procedure is as follows: First, the gas sensor module is assembled, and the transparent observation cover is securely installed. After the sensor resistance stabilizes, as observed through the software, 1 mL of DI water is injected into the evaporation platform (120℃) using a calibrated syringe. When a temporary plateau appears on the humidity curve, assess the situation and repeat the injection if necessary. The experimental setup is illustrated in the accompanying figure. These details have been incorporated into the support information for clarity and completeness.


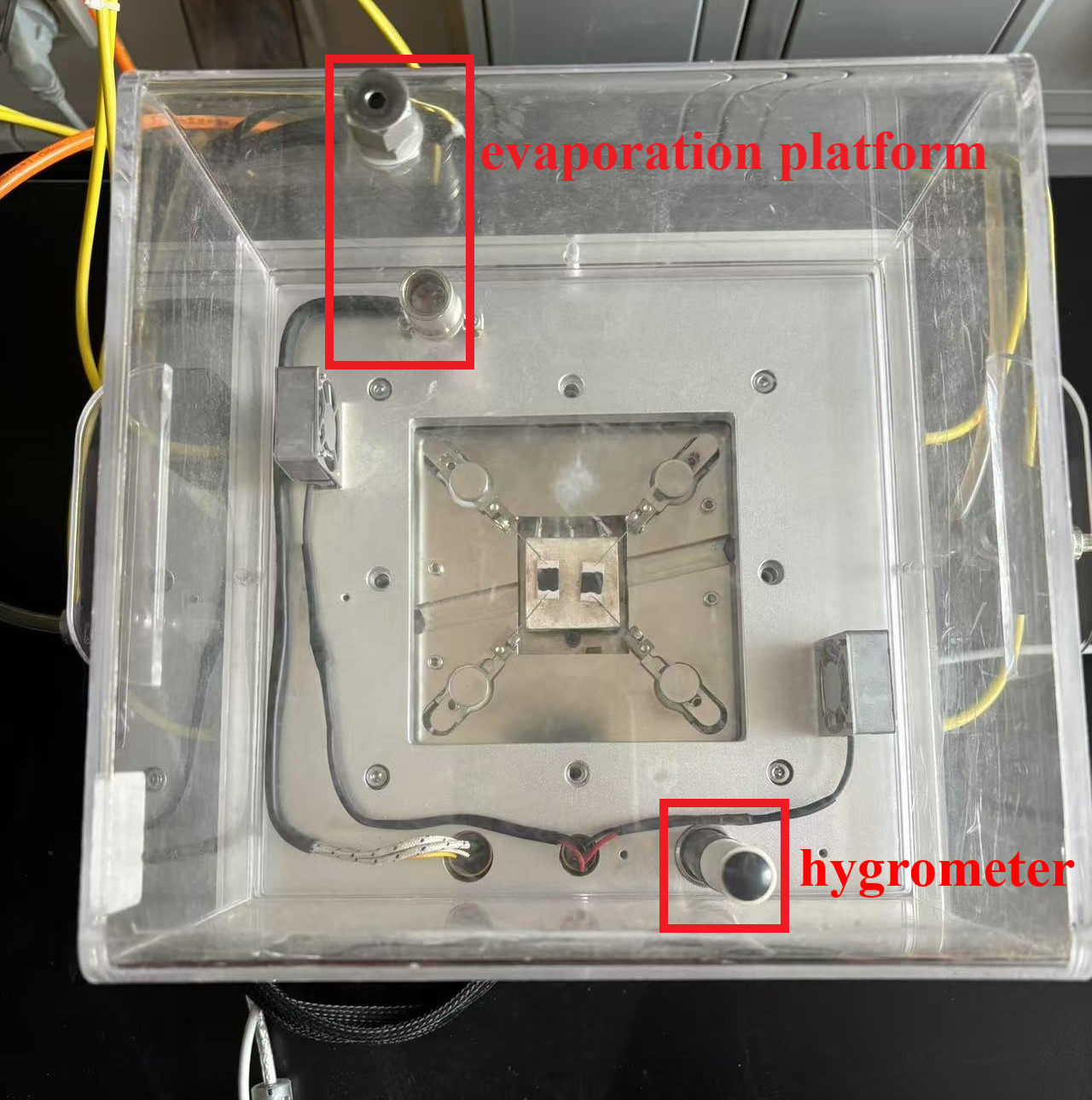


First-principles calculations were carried out using the VASP code^[1,2]^. A GGA type Perdew Burke-Ernzerhof (PBE)^[3,4]^ functional to define the exchange-correlation. Dispersion correction to the energy using Grimme's DFT-D3 scheme is included ^[5,6]^. Projector augmented-wave (PAW) pseudopotentials^[7]^ for all atoms were used for valence-core interactions. A high kinetic energy cutoff of 500 eV was chosen for the plane wave basis set expansion. A 10^-5^ eV break condition were considered in the self-consistent field calculations. Atomic positions and lattice constants were allowed to relax using a conjugate gradient algorithm until all Hellman-Feynman forces are smaller than 0.05 eV/Å. A 2×2×1 centered k-point mesh was chosen for Brillouin-zone sampling. The structures were modeled by a periodic slab that separated by a 15 Å of vacuum in the z axis direction.

The adsorption energy (E_ads_) is calculated via the following formula:

E_ads_ = E_total_ – E_slab_ – E_TEA_

where E_total_, E_slab_ and E_TEA_ represent the DFT energies of adsorbates with substrates, the substrates and TEA gas adsorbates, respectively. The more negative this E_ads_ value is, the stronger the adsorption.


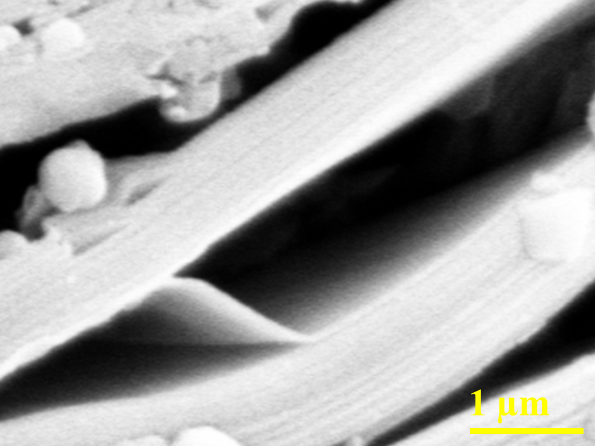


**Figure S1** Scanning electron microscopy (SEM) images of MXene


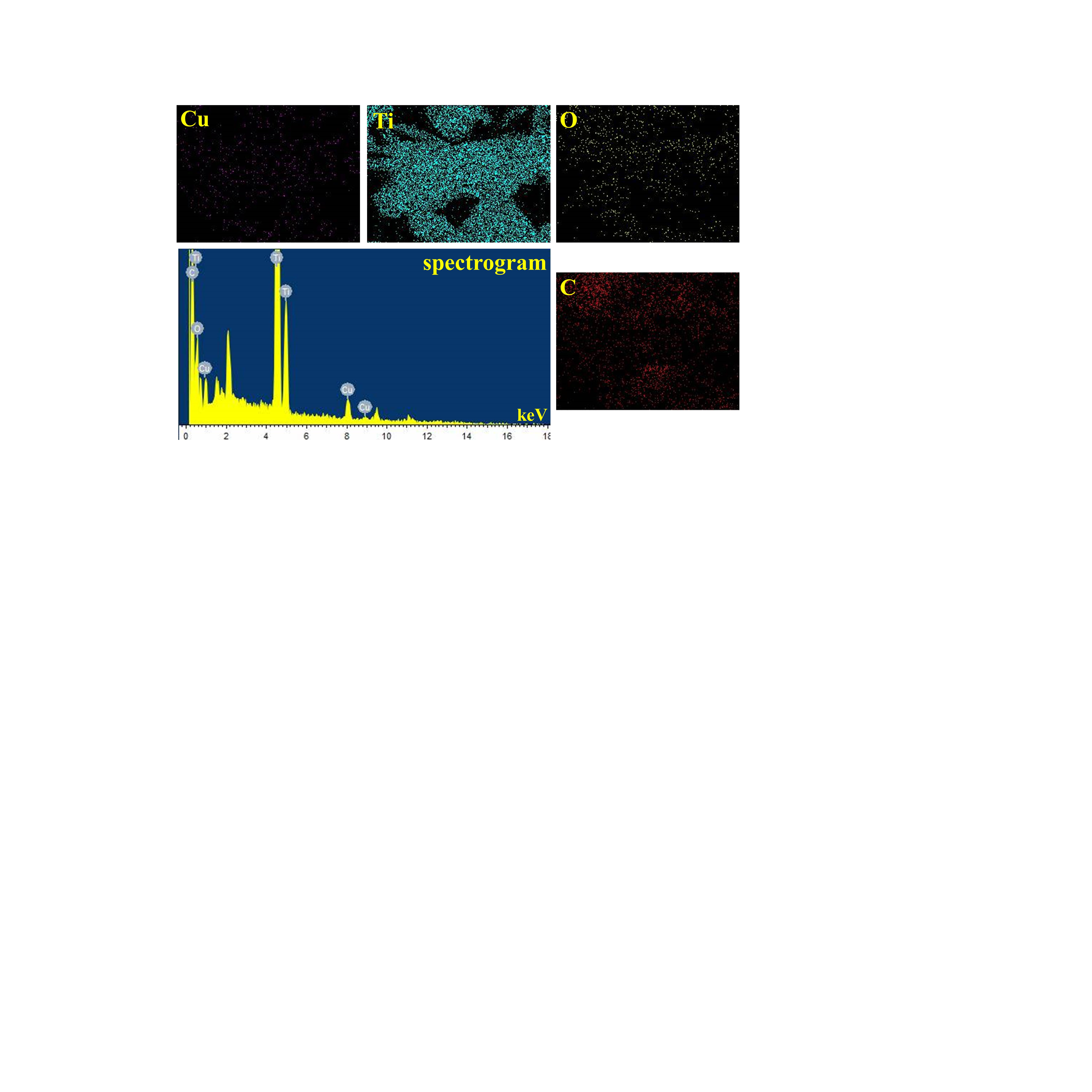


**Figure S2** Elemental mapping of Cu-MXene





**Figure S3** BET of MX@Cu-T





**Figure S4** EIS spectra of MXene, Cu-HHTP-8 and MX@Cu-8

**Table S1.** Binding energy of the XPS Ti 2p spectrum of MXene and MX@Cu.

| BE (eV)  Samples | Ti-C | Ti-F | Ti-O |
| --- | --- | --- | --- |
| MXene | 459.1(456) | 463.3(457.9) | 462(456.9) |
| MX@Cu | 458.9(455.5) | 464.6(457.1) | 461.8(456.4) |

**Table S2.** Binding energy of the XPS C 1s spectrum of MXene, Cu-HHTP and MX@Cu.

| BE (eV)  Samples | π-π* | C=O | C-O | C-C | C-Ti-O | C-Ti |
| --- | --- | --- | --- | --- | --- | --- |
| MXene | - | 288.4 | 286.2 | 284.9 | 283.6 | 282.1 |
| Cu-HHTP | 289.9 | 288.2 | 286.3 | 284.4 | - | - |
| MX@Cu | 289.7 | 288.0 | 286.1 | 284.4 | - | 282.2 |





**Figure S5** The resistances for MX@Cu-2 in the TEA concentration range of 1-200 ppm under room temperature.





**Figure S6** The resistances for MX@Cu-16 in the TEA concentration range of 1-200 ppm under room temperature.





**Figure S7** The resistances for Cu-HHTP in the TEA concentration range of 1-200 ppm under room temperature.





**Figure S8** The response value and concentration linear correlation of MX@Cu-2 in TEA.





**Figure S9** The response value and concentration linear correlation of MX@Cu-16 in TEA.





**Figure S10** The response value and concentration linear correlation of Cu-HHTP in TEA.





**Figure S11** The duration required for the response and recovery of MX@Cu-2 when exposed to 50 ppm TEA.





**Figure S12** The duration required for the response and recovery of MX@Cu-16 when exposed to 50 ppm TEA.





**Figure S13** The duration required for the response and recovery of Cu-HHTP when exposed to 50 ppm TEA.





**Figure S14** Response/recovery times of the five repeated tests when exposed to 20 ppm TEA for MX@Cu-2





**Figure S15** Response/recovery times of the five repeated tests when exposed to 20 ppm TEA for MX@Cu-16





**Figure S16** Response/recovery times of the five repeated tests when exposed to 20 ppm TEA for Cu-HHTP





**Figure S17** The response value (%) of MX@Cu-8 towards VOCs at a concentration of 100 ppm.

*

*

**Figure S18** Histogram of adsorption energy of Cu, C and O on Cu-HHTP


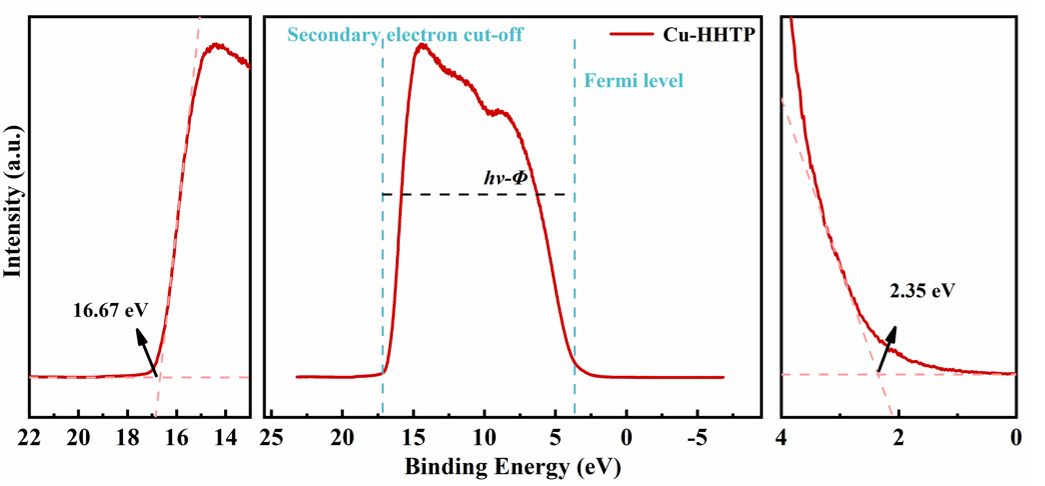


**Figure S19** UPS spectrum of the Cu-HHTP


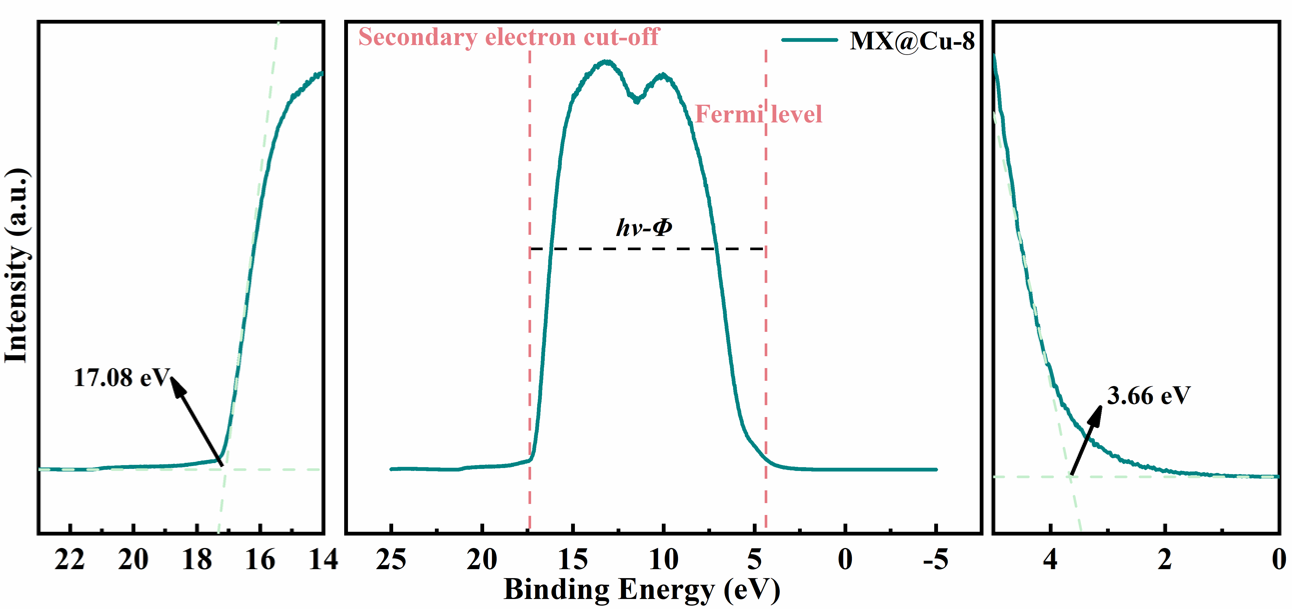


**Figure S20** UPS spectrum of the MX@Cu-8





**Figure S21** UV–vis absorbance spectra and estimated band gap values of MXene, Cu-HHTP and MX@Cu-8





**Figure S22** Response/recovery times of the five repeated tests when exposed to 200 ppm TEA for MXene





**Figure S23** The resistances for MX@Cu-8 in the 100 ppm EtOH at room temperature.





**Figure S24** The resistances for MX@Cu in the meat freshness detection under room temperature.

**Table S3** Comparison of gas sensitive properties of different materials to TEA

| Sensing materials | Temp. (℃) | Conc. (ppm) | τ_res_ (s) | Ref. |
| --- | --- | --- | --- | --- |
| WO_3_/SnO_2_ | 220 | 1 | 6 | ^[8]^ |
| ZnO/Co_3_O_4_ | 100 | 5 | 30 | ^[9]^ |
| Pd/In_2_O_3_ | 220 | 1 | 108 | ^[10]^ |
| CuCrO_2_ | 100 | 100 | 90 | ^[11]^ |
| Fe/NiO | 260 | 1 | 26 | ^[12]^ |
| WO_3_ nanorods | 250 | 1 | 45 | ^[13]^ |
| Carborized polymer dots/ WO_3_ | 140 | - | 63 | ^[14]^ |
| Cu-HHTP-8 | RT | 1 | 16 | **Figure S3** |
| MX@Cu-8 | RT | 1 | 4 | This work |

**Table S4** Comparison of gas sensitive properties of different cMOF materials

| Sensing materials | Temp. (℃) | Gas | Conc.(ppm) | Response | τ_res_/τ_rec_ | LOD | moisture resistance | Ref. |
| --- | --- | --- | --- | --- | --- | --- | --- | --- |
| AF/Cu-CAT | RT | NH_3_ | 10 | ~211% | 67 / 43 s | 0.5 ppm | <65%RH | ^[15]^ |
| (with UV-light irradiation) |  |  |  | ~235% | 4.8 / 7.5 s |  | 11%RH to 97%RH declines ~12 % |  |
| Cu_3_HIB_2_ | RT | CO_2_ | 100 | −0.62% | 7-8 / 10-11 min | 67 ppm | <80%RH | ^[16]^ |
| Pd@Cu_3_(HHTP)_2_ | RT | NO_2_ | 1 | −13.5% | 13.8 min / not rec. | 1 ppm | - | ^[17]^ |
| Pt@Cu_3_(HHTP)_2_ |  |  |  | −12.1% | 14 min / not rec. |  | - |  |
| Cu_3_(HHTP)_2_ |  |  |  | −5.0% | 18 min / not rec. |  | - |  |
| Zn-HHTP | RT | CO | 30 | - | 56 s / 150 s | 3.96 ppm | <40%RH | ^[18]^ |
| Cu_3_(HHTP)_2_ | RT | MeOH | 200 | ~-9.5% | 30 s / - | - | - | ^[19]^ |
| Cu_3_(HITP)_2_ |  | Benzene |  | ~-2.7% |  |  | - |  |
| Ni_3_(HITP)_2_ |  |  |  | ~-5.9% |  |  | - |  |
| Bi(HHTP) | RT | NO | 40 | −54.8 ± 6% | 15 min / not rec. | 0.15 ppm | - | ^[20]^ |
|  |  | NH_3_ |  | 58.4 ± 2% | 15 min / not rec. | 0.29 ppm | - |  |
| CoNiHHTP | RT under 405 nm irradiation | NO_2_ | 1×10^-3^ | 15.6% | 3.6 min / 2.7 min | - | <52%RH | ^[21]^ |
| Cu_3_(HITP)_2_ | RT | NH_3_ | 10 | ~3% | - | <0.5 ppm | <60%RH | ^[22]^ |
| Cu-TCPP-10C-on-Cu-HHTP-20C | RT | benzene | 0.12 | 10% | 1.53 / 10.72 min | 0.12 ppm | - | ^[23]^ |
| Cu_3_HHTP_2_ | RT | NH_3_ | 80 | 0.7 ± 0.3 % | 5 min / 10 min | - | - | ^[24]^ |
|  |  |  |  | - |  |  | - |  |
|  |  | NO |  | 1.8 ± 0.4 % |  |  | - |  |
| Ni_3_HHTP_2_ |  |  |  | 1.7 ± 1.0 % |  |  | - |  |
|  |  | H_2_S |  | 0.5 ± 0.3 % |  |  | - |  |
|  |  |  |  | 4.2 ± 1.0 % |  |  | - |  |
| M_3_HHTP_2_/graphite, (M = Fe, Co, Ni and Cu) | RT | NH_3_ | 80 | 2.1 ± 0.6% | - | 19 ppm | - | ^[25]^ |
|  |  | NO |  | −1.8 ± 0.4% |  | 17 ppm | - |  |
|  |  | H_2_S |  | 1.0 ± 0.5% |  | 35 ppm | - |  |
| Cu_3_(HHTP)(THQ) | RT | NH_3_ | 10 | ~3.5% | 1.65 / 2.57 min | ~0.02 ppm |  | ^[26]^ |
| Cu_3_HHTT_2_-1 | RT | NH_3_ | 60 | S= −161.6 | / | - | 10%RH | ^[27]^ |
|  |  | NO_2_ | 3 | S = 104.5 |  |  | 50%RH |  |
| Cu_3_(HHTP)_2_ | RT | NH_3_ | 100 | 129% | 1.36 / 9.11 min | 0.5 ppm | - | ^[28]^ |
| HITP doped Cu-HHTP-10C | RT | NH_3_ | 100 | ~100% | < 1 min / < 10 min | 0.024-0.096 ppm | - | ^[29]^ |
| Cu-HHTP-20 | RT | NH_3_ | 100 | 161% | 35 s / 15 min | ~87 ppt | - | ^[30]^ |
| Cu-MOF/PVA/IL | RT | H_2_S | 1 | ~96-99% | 12 s / - | 1 ppm | - | ^[31]^ |
| Ni_x_Cu_3_-x(HHTP)_2_/PPy NCPs | RT | H_2_S | 10 | ~0.27 | 81 s / 568 s | 500 ppb | <40%RH | ^[32]^ |
| Ni_3_HHTP_2_ | RT | NO | 80 | −49% ± 10% | ~25 min / not rec. | 0.16 ppm | <60%RH | ^[33]^ |
|  |  |  |  | 81% ± 6% | ~20 min / not rec. | 1.4 ppm |  |  |
| Ni_3_HITP_2_ |  | H_2_S |  | = 98% ± 9% | ~15 min / not rec. | 0.52 ppm |  |  |
|  |  |  |  | = 97% ± 2% | ~22 min / not rec. | 0.23 ppm |  |  |
| LIG@Cu_3_HHTP_2_ | RT | NO_2_ | 1×10^-2^ | ~1 | 16 s / 15 s | 0.168 ppb |  | ^[34]^ |
| Cu-TCPP-Cu-12 | RT | benzene | 1 | -20 Hz | 8 / 11s | 65 ppb | - | ^[35]^ |
| Fe_2_O_3_-Cu_3_(HHTP)_2_-NFs | RT | NO_2_ | 4×10^-2^ | 30.4% | dark~10 min / not rec.  blue~30 / 130 min | 11 ppb | <50%RH | ^[36]^ |
| MOF@SnS_2_-12 | RT | NH_3_ | 5 | ~10.2% | <5 min / <5 min | 9.84 ppb | - | ^[37]^ |
| AF/ZnO/Zn_3_ (HHTP)_2_ | 50 | TEA | 0.5 | 120% | ~ 56 / 128 s | 120 ppb | <33%RH | ^[38]^ |
| 2D-kgm-Cu-MOF-3 | RT | NH_3_ | 100 | - | 3.62 / 8.95 min | 10.8 ppb | - | ^[39]^ |
| MX@Cu-8 | RT | TEA | 50 | 11.32% | 4 / 187 s | <1 ppm | <80%RH | This work |

**References**

[1] G. Kresse, J. Furthmüller, *Computational Materials Science* 1996, *6*, 15.

[2] G. Kresse, J. Furthmüller, *Phys. Rev. B* 1996, *54*, 11169.

[3] J. P. Perdew, A. Ruzsinszky, G. I. Csonka, O. A. Vydrov, G. E. Scuseria, L. A. Constantin, X. Zhou, K. Burke, *Phys. Rev. Lett.* 2008, *100*, 136406.

[4] J. P. Perdew, K. Burke, M. Ernzerhof, *Phys. Rev. Lett.* 1996, *77*, 3865.

[5] S. Grimme, *Journal of Computational Chemistry* 2006, *27*, 1787.

[6] S. Grimme, J. Antony, S. Ehrlich, H. Krieg, *The Journal of Chemical Physics* 2010, *132*, 154104.

[7] P. E. Blöchl, *Phys. Rev. B* 1994, *50*, 17953.

[8] V. K. Tomer, S. Devi, R. Malik, S. P. Nehra, S. Duhan, *Sensors and Actuators B: Chemical* 2016, *229*, 321.

[9] Y. Xiong, W. Liu, X. Qiao, X. Song, S. Wang, X. Zhang, X. Wang, J. Tian, *Sensors and Actuators B: Chemical* 2021, *346*, 130486.

[10] X. Liu, K. Zhao, X. Sun, C. Zhang, X. Duan, P. Hou, G. Zhao, S. Zhang, H. Yang, R. Cao, X. Xu, *Sensors and Actuators B: Chemical* 2019, *285*, 1.

[11] H. Liu, X. Cao, H. Wu, B. Li, Y. Li, W. Zhu, Z. Yang, Y. Huang, *Sensors and Actuators B: Chemical* 2020, *324*, 128743.

[12] D. Wang, C. Zhai, L. Du, K. Gu, M. Zhang, *Inorg. Chem. Front.* 2020, *7*, 1474.

[13] Q. Hu, J. He, J. Chang, J. Gao, J. Huang, L. Feng, *ACS Appl. Nano Mater.* 2020, *3*, 9046.

[14] M. Zhang, Z. Zhao, B. Hui, J. Sun, J. Sun, W. Tian, Z. Zhang, K. Zhang, Y. Xia, *Journal of Hazardous Materials* 2021, *416*, 126161.

[15] K. Liu, W. Tian, B. Hui, K. Zhang, Y. Xia, *Materials Science and Engineering: R: Reports* 2024, *160*, 100827.

[16] I. Stassen, J.-H. Dou, C. Hendon, M. Dincă, *ACS Cent. Sci.* 2019, *5*, 1425.

[17] W. Koo, S. Kim, J. Jang, D. Kim, I. Kim, *Advanced Science* 2019, *6*, 1900250.

[18] M. S. More, G. A. Bodkhe, F. Singh, Babasaheb. N. Dole, M.-L. Tsai, T. Hianik, M. D. Shirsat, *Synthetic Metals* 2023, *296*, 117357.

[19] M. G. Campbell, S. F. Liu, T. M. Swager, M. Dincă, *J. Am. Chem. Soc.* 2015, *137*, 13780.

[20] A. Aykanat, C. G. Jones, E. Cline, R. M. Stolz, Z. Meng, H. M. Nelson, K. A. Mirica, *ACS Appl. Mater. Interfaces* 2021, *13*, 60306.

[21] R. Xu, B. Sun, W. Ji, J. Sun, P. Li, Z. Ren, L. Jing, *ACS Sens.* 2024, *9*, 3187.

[22] M. G. Campbell, D. Sheberla, S. F. Liu, T. M. Swager, M. Dincă, *Angew Chem Int Ed* 2015, *54*, 4349.

[23] M. Yao, J. Xiu, Q. Huang, W. Li, W. Wu, A. Wu, L. Cao, W. Deng, G. Wang, G. Xu, *Angew Chem Int Ed* 2019, *58*, 14915.

[24] M. K. Smith, K. E. Jensen, P. A. Pivak, K. A. Mirica, *Chem. Mater.* 2016, *28*, 5264.

[25] M. Ko, A. Aykanat, M. Smith, K. Mirica, *Sensors* 2017, *17*, 2192.

[26] M. Yao, J. Zheng, A. Wu, G. Xu, S. S. Nagarkar, G. Zhang, M. Tsujimoto, S. Sakaki, S. Horike, K. Otake, S. Kitagawa, *Angew Chem Int Ed* 2020, *59*, 172.

[27] Y.-M. Jo, D.-H. Kim, J. Wang, J. J. Oppenheim, M. Dincă, *J. Am. Chem. Soc.* 2024, *146*, 20213.

[28] M. Yao, X. Lv, Z. Fu, W. Li, W. Deng, G. Wu, G. Xu, *Angewandte Chemie* 2017, *129*, 16737.

[29] A.-Q. Wu, W.-Q. Wang, H.-B. Zhan, L.-A. Cao, X.-L. Ye, J.-J. Zheng, P. N. Kumar, K. Chiranjeevulu, W.-H. Deng, G.-E. Wang, M.-S. Yao, G. Xu, *Nano Res.* 2021, *14*, 438.

[30] Y. Lin, W. Li, Y. Wen, G. Wang, X. Ye, G. Xu, *Angew Chem Int Ed* 2021, *60*, 25758.

[31] A. Ali, H. H. D. AlTakroori, Y. E. Greish, A. Alzamly, L. A. Siddig, N. Qamhieh, S. T. Mahmoud, *Nanomaterials* 2022, *12*, 913.

[32] X. Lang, X. Xing, X. Zhao, L. Du, Y. Tian, Z. Li, X. Chen, H. Fan, D. Yang, *Sensors and Actuators B: Chemical* 2024, *401*, 135020.

[33] M. K. Smith, K. A. Mirica, *J. Am. Chem. Soc.* 2017, *139*, 16759.

[34] H. Lim, H. Kwon, H. Kang, J. E. Jang, H.-J. Kwon, *Nat Commun* 2023, *14*, 3114.

[35] Z. Ma, Y. Zhang, Z. Xue, Y. Fan, L. Wang, H. Wang, A. Zhong, J. Xu, *ACS Sens.* 2024, *9*, 1906.

[36] Y.-M. Jo, K. Lim, J. W. Yoon, Y. K. Jo, Y. K. Moon, H. W. Jang, J.-H. Lee, *ACS Cent. Sci.* 2021, *7*, 1176.

[37] Y. Huang, X. Zhang, S. Liu, R. Wang, J. Guo, Y. Chen, X. Ma, *Chemical Engineering Journal* 2023, *458*, 141364.

[38] K. Liu, M. Zhang, X. Du, A. Zhou, B. Hui, Y. Xia, K. Zhang, *Nano Res.* 2023, *16*, 1296.

[39] Z. Shan, J. Xiao, M. Wu, J. Wang, J. Su, M. Yao, M. Lu, R. Wang, G. Zhang, *Angew Chem Int Ed* 2024, *63*, e202401679.

1. * Corresponding authors, E-mail addresses: zhijun_ding@139.com (Zhi-jun Ding), ylmyl@163.com (Lingmin Yu). [↑](#footnote-ref-1)
